# Obesity, Fat Mass and Immune System: Role for Leptin

**DOI:** 10.3389/fphys.2018.00640

**Published:** 2018-06-01

**Authors:** Vera Francisco, Jesús Pino, Victor Campos-Cabaleiro, Clara Ruiz-Fernández, Antonio Mera, Miguel A. Gonzalez-Gay, Rodolfo Gómez, Oreste Gualillo

**Affiliations:** ^1^The NEIRID Group (Neuroendocrine Interactions in Rheumatology and Inflammatory Diseases), Servizo Galego de Saude and Instituto de Investigación Sanitaria de Santiago, Santiago University Clinical Hospital, Santiago de Compostela, Spain; ^2^Servizo Galego de Saude, Division of Rheumatology, Santiago University Clinical Hospital, Santiago de Compostela, Spain; ^3^Epidemiology, Genetics and Atherosclerosis Research Group on Systemic Inflammatory Diseases, Hospital Universitario Marqués de Valdecilla, Universidad de Cantabria and IDIVAL, Santander, Spain; ^4^Musculoskeletal Pathology Group, Servizo Galego de Saude and Instituto de Investigación Sanitaria de Santiago, Santiago University Clinical Hospital, Santiago de Compostela, Spain

**Keywords:** adipokines, adipose tissue, immunometabolism, leptin, metabolism, rheumatic diseases, rheumatoid arthritis, Type 2 diabetes mellitus (T2DM)

## Abstract

Obesity is an epidemic disease characterized by chronic low-grade inflammation associated with a dysfunctional fat mass. Adipose tissue is now considered an extremely active endocrine organ that secretes cytokine-like hormones, called adipokines, either pro- or anti-inflammatory factors bridging metabolism to the immune system. Leptin is historically one of most relevant adipokines, with important physiological roles in the central control of energy metabolism and in the regulation of metabolism-immune system interplay, being a cornerstone of the emerging field of immunometabolism. Indeed, leptin receptor is expressed throughout the immune system and leptin has been shown to regulate both innate and adaptive immune responses. This review discusses the latest data regarding the role of leptin as a mediator of immune system and metabolism, with particular emphasis on its effects on obesity-associated metabolic disorders and autoimmune and/or inflammatory rheumatic diseases.

## Introduction

Obesity, the greater public health problem in the western world, is associated with high-incident chronic autoimmune and inflammatory pathologies, such as T2DM, NAFLD, OA, and RA, thus having a huge social and economic impact (Zhang et al., 2014). Adipose tissue, initially considered as a simple energy storage tissue, is now recognized as an active endocrine organ and a *bona fide* immune organ, constituted not only by adipocytes but also by fibroblasts, endothelial cells and a wide array of immune cells (adipose tissue macrophages, neutrophils, mast cells, eosinophils, T and B cells that maintains tissue homeostasis in lean individuals (Huh et al., 2014; Vieira-Potter, 2014). The adipocyte expansion caused by positive energy balance leads to adipocyte hypoxia, apoptosis, and cell stress, ultimately resulting in the expression of chemoattractant molecules and infiltration of inflammatory cells (Vieira-Potter, 2014). The obese adipose tissue is also characterized by a markedly deregulated production of adipose tissue-derived factors, i.e., adipokines, a growing family of low molecular weight, biologically active proteins with pleiotropic functions (Al-Suhaimi and Shehzad, 2013). Adipokines are crucial players not only in energy metabolism but also in inflammation and immunity, most of them being increased in obesity and contributing to the associated ‘low-grade inflammatory state’ (Tilg and Moschen, 2006).

**FIGURE 1:**
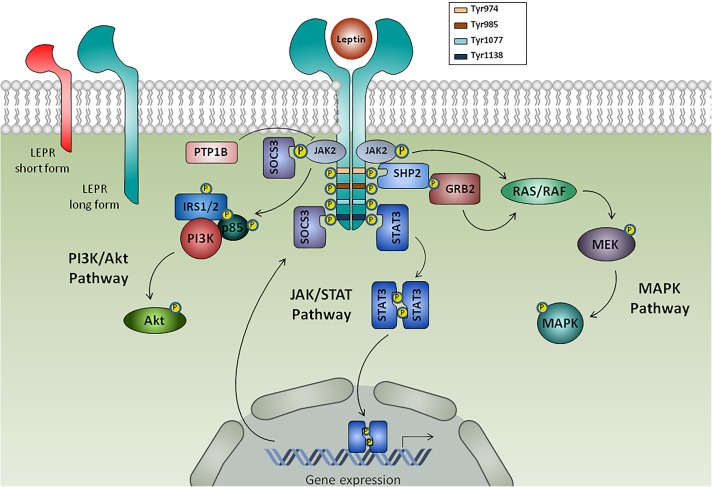
Leptin receptors and intracellular leptin signaling pathways. Leptin binds to its receptor (LEPR) isoforms: the soluble isoform (not shown), the short isoform and the long isoform. Binding of leptin to the long form of LEPR results in its dimerization and prompts Janus kinase 2 (JAK2) autophosphorylation, which phosphorylates cytoplasmatic domain of LEPR in tyrosine residues (Tyr974, Tyr985, Tyr1077, Tyr1138), each one functioning as docking sites for cytoplasmic adaptors. LEPR-phosphorylated Tyr1138 mediates the interaction with signaling transducer and activator of transcription 3 (STAT3), which dimerize and translocate to the nucleus to activate gene transcription of target genes, such as suppressor of cytokine signaling 3 (SOCS3) that acts as a negative feedback signaling. Additionally, leptin induces the activation of SHP2, which then recruits the adaptor protein Grb2 to prompt activation of Ras/Raf/MAPK signaling cascade. Leptin also mediated phosphatidylinositol-3-kinase (PI3K)/Akt activation via insulin receptor substrate 1/2 (IRS1/2) and protein tyrosine phosphatase 1B (PTP1B) acts as a negative regulator of leptin signaling through JAK2 dephosphorylation.

**FIGURE 2:**
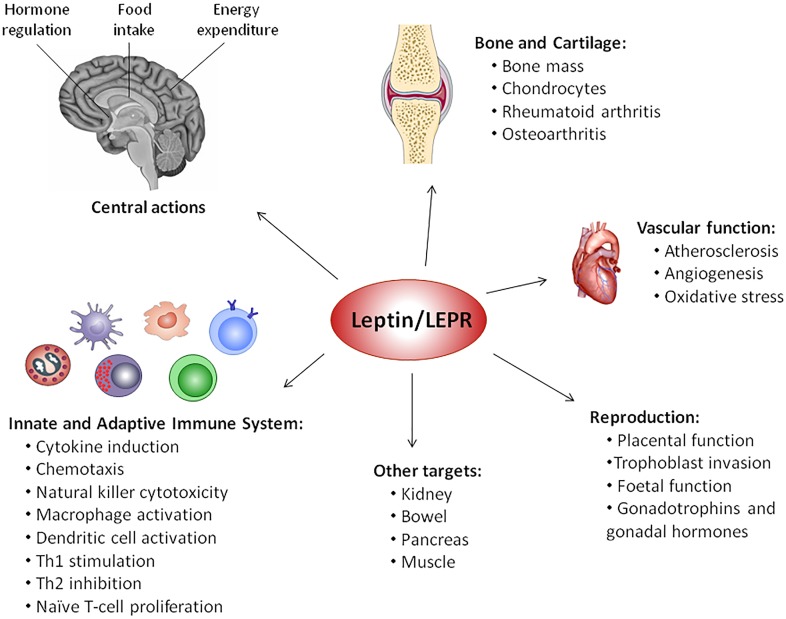
Pleiotropic nature of leptin. Since its discovery in 1994, several physiological functions have been attributed to leptin, such as modulation of vascular function, reproduction, bone metabolism, inflammation, infection, and immune responses, behind central regulation of food intake, energy expenditure and hormone regulation, via activation of leptin receptor (LEPR).

Leptin was discovered in 1994 by the group of Jeffrey Friedman (Zhang et al., 1994) and is the best-characterized member of adipokine family. Encoded by *LEP* gene (the human homolog of murine *ob* gene), leptin is a 16 kDa non-glycosylated protein mainly produced by adipocytes, but also by skeletal muscle, intestine, brain, joint tissues and bone (Scotece et al., 2014). This adipokine exerts its physiological activity through its receptor (LEPR or Ob-R), a class I cytokine receptor family from diabetes (*db*) gene (Münzberg and Morrison, 2015). There are at least six LEPR isoforms that differ in the length of the cytoplasmic domain: a soluble isoform, four short isoforms, and a long isoform, which has the full intracellular domain that allows the transduction of leptin signal via JAK and STAT signaling pathways (Frühbeck, 2006). Alternatively to canonical JAK/STAT pathway, LEPR could activate ERK 1/2, p38 MAPK, JNK, PKC, and PI3K/Akt pathways (Zhou and Rui, 2014) (**Figure 1**). This hormone, together with other regulatory molecules, has a central role in appetite and body weight homeostasis by inducing anorexigenic factors (as cocaine-amphetamine-related transcript) and suppressing orexigenic neuropeptides (as neuropeptide Y) on hypothalamus (Al-Suhaimi and Shehzad, 2013; Rosenbaum and Leibel, 2014). Therefore, central leptin resistance, caused by impairment of leptin transportation, leptin signaling and leptin target neural circuits, is considered the main risk factor for the obesity pathogenesis.(Al-Suhaimi and Shehzad, 2013; Rosenbaum and Leibel, 2014). Interestingly, leptin release is modulated in a circadian rhythm manner, which has been correlated with sweet taste recognition (Nakamura et al., 2008). Moreover, leptin also affects other physiological functions, namely bone metabolism, inflammation, infection and immune responses (Scotece et al., 2014) (**Figure 2**). Accordingly, LEPR is expressed in across the cells of innate and adaptive immune system, evoking leptin as a crucial linker of neuroendocrine and immune systems (Carlton et al., 2012; Procaccini et al., 2017).

This review summarizes the latest data regarding the role of leptin as a mediator of innate and adaptive immune cells activity, and its effects on obesity-associated metabolic disorders, namely T2DM and NAFLD, and autoimmune and/or inflammatory rheumatic diseases, such as OA and RA.

## Leptin and Immunometabolism

The rising prevalence of obesity in western society is paralleled with a significant augment in autoimmune diseases. Accordingly, numerous association studies had demonstrated that overweight is implicated in a higher risk of developing multiple sclerosis (Kavak et al., 2015), RA (Ajeganova et al., 2013), and psoriasis (Duarte et al., 2013). On the other hand, malnutrition/starvation has been long related to increased susceptibility to infectious diseases (Taylor et al., 2013; Jones et al., 2014). These observations bring out immune response as a highly energy-dependent biological process that is dependent on an adequate food intake and metabolism. In fact, a recent article critically discuss the correlation between autoimmunity and overnutrition or metabolic pressure (De Rosa et al., 2017). At the interface of the historically distinct fields of immunology and metabolism, immunometabolism has emerged as a new research discipline (Mathis and Shoelson, 2011). In the last years, it has been evidenced that metabolic status of immune cells directly determines their function and differentiation, thus affecting immunity and tolerance, as well as the failure of the immune response in autoimmune pathologies (Gaber et al., 2017). In fact, innate and adaptive immune cells adapt to altered tissue microenvironment, characterized by hypoxia and nutrient competition, by reprogramming their metabolism (Gaber et al., 2017), and failure in this metabolic reconfiguration ultimately leads to a deregulated immune response and pathology (Gaber et al., 2017).

Leptin, the forerunner of adipokine family, is a key sensor of energy metabolism and a cornerstone in the regulation of metabolism-immune system interplay. Malnutrition results in hypoleptinemia, while obesity leads to hyperleptinemia, both conditions affecting the immune response in an opposite manner. In particular, obese subjects demonstrated decreased levels of Treg (central players in the control of peripheral immune tolerance), which are inversely correlated with leptin levels and BMI (Matarese et al., 2010). In malnutrition, altered T cell function and metabolism was associated with decreased leptin levels (Cohen et al., 2017). *Leptin* and *LepR*-deficient mouse models presented augmented number and activity of Treg cells together with a resistance to autoimmune diseases, and leptin replacement rescues Treg cell levels to wild-type mice values (Matarese et al., 2010). Accordingly, human T cell activation and production of cytokines can be induced after incubation with 10 ng/mL exogenous leptin following nutritional rehabilitation (Rodríguez et al., 2007). Furthermore, the central effect of leptin on the hypothalamus is mediated, at least in part, by inhibition of hypothalamic-pituitary-adrenal axis and activation of the sympatho-adrenal axis, having the sympathetic nervous system a function in the central control of the immune system (Pérez-Pérez et al., 2017). Moreover, most immune cells express LEPR at their surface, which evokes a straight action of leptin in the modulation of the immune response (Procaccini et al., 2017).

## Leptin and Innate Immunity (**Figure 3**)

**FIGURE 3:**
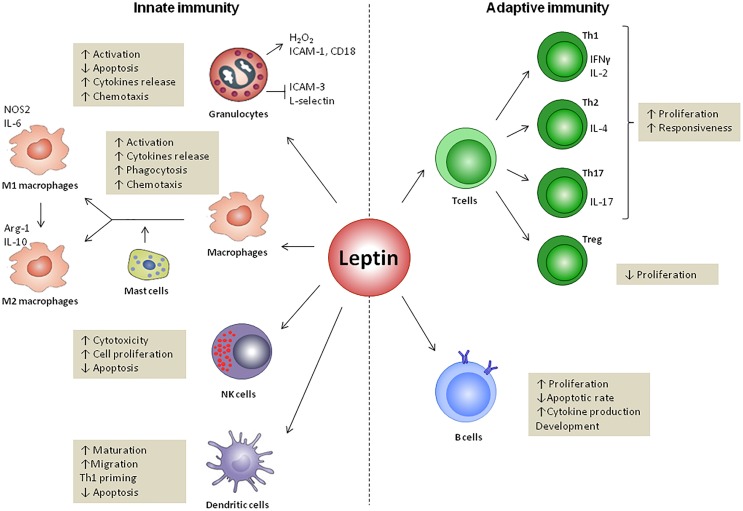
Leptin effects on innate and adaptive immunity. Leptin regulates both innate and adaptive responses through modulation of immune cells survival and proliferation as well as its activity. In innate immunity, leptin increases the cytotoxicity of natural killer (NK) cells and promotes the activation of granulocytes, macrophages and DCs. Leptin also regulates the M1- or M2-phenotype polarization and modulates DCs, licensing them towards type 1 T helper cells (Th1) priming. In adaptive immunity, leptin increases the proliferation of naïve T cells and B cells while it reduces that of regulatory T cells (Treg). Leptin promotes the switch towards a pro-inflammatory Th1 (which secretes IFNγ) rather than anti-inflammatory Th2 (which secretes IL-4) phenotype, and facilitates Th17 responses. Finally, leptin activates B cells to secrete cytokines and modulates B cell development.

### Granulocytes (Neutrophils, Eosinophils, and Basophils)

Human polymorphonuclear neutrophils express LEPR (Caldefie-Chezet et al., 2001), but only the short-form (Ob-Ra) was been detected (Zarkesh-Esfahani et al., 2004). Although the short-form of LEPR lacks most of the intracellular domain of the receptor, it is enough to signal through MAPK pathways, enhancing CD 11b expression and preventing apoptosis, but not through JAK-STAT pathways as long-form of LEPR (Bjorbaek et al., 1997; Zarkesh-Esfahani et al., 2004). Leptin is likely to act as a survival cytokine for neutrophils. At 500 nM, leptin delayed the cleavage of Bid and Bax, mitochondrial release of cytochrome C and second mitochondria-derived activator of caspase, as well as the activation of caspase-3 and caspase-8 (Bruno et al., 2005). PI3K, NF-κB, and MAPK pathways were involved in the anti-apoptotic activity of leptin in human neutrophils *in vitro* (Bruno et al., 2005; Sun et al., 2013). Additionally, leptin (250 ng/ml) stimulated the release of oxygen radicals, such as superoxide anion and hydrogen peroxide, by PMNs (Caldefie-Chezet et al., 2001, 2003).

There is strong evidence for an effect of leptin on neutrophil chemotaxis and infiltration. Leptin (50 ng/ml) mediated the migration of human neutrophils *in vitro*, through activation of p38 MAPK and Src kinases (Montecucco et al., 2006), and by indirect mechanisms via TNF-α released by monocytes (Caldefie-Chezet et al., 2001; Zarkesh-Esfahani et al., 2004), having no secretagogue properties (no detectable [Ca^2+^]i mobilization, oxidant production, or β2-integrin upregulation) (Montecucco et al., 2006). Otherwise, leptin inhibits neutrophil chemotaxis to classical chemoattractants, like interleukin (IL)-8 (Montecucco et al., 2006). Murine neutrophils with Q223R LEPR mutation had reduced chemotaxis toward leptin (Naylor et al., 2014), while neutrophils from human volunteers and wild-type C57BL/6 mice migrated toward leptin in a dose-dependent manner, requiring JAK2/PI3K signaling (Ubags et al., 2014). Nevertheless, one study found that physiological concentrations of leptin (1–100 ng/ml) do not affect human neutrophils, and high leptin concentrations induced survival and changes in neutrophils proteome, but no effect on chemotaxis was observed (Kamp et al., 2013). *In vivo* studies clarified the effect of leptin in neutrophils. It was observed that neutrophil populations were enhanced in rats with high-fat-diet induced obesity, compared with control diet rats (do Carmo et al., 2013), and neutrophils from obese subjects displayed elevated superoxide release and chemotactic activity (Brotfain et al., 2015). Furthermore, leptin administration (50 μg) increased pulmonary neutrophilia in *Escherichia coli* pneumonia murine model as well as in healthy mice (Ubags et al., 2014).

Alike neutrophils, both human eosinophils and basophils expressed LEPR on the cell surface (Bruno et al., 2005; Suzukawa et al., 2011). In eosinophils, leptin (50 ng/ml) enhanced the release of pro-inflammatory cytokines IL-1β and IL-6, and chemokines IL-8, growth-related oncogene-α and MCP-1 (Wong et al., 2007). It also modulated the surface expression of adhesion molecules; in particular, up-regulates ICAM-1 and CD18, and suppress ICAM-3 and L-selectin (Wong et al., 2007). Treatment of human eosinophils with recombinant leptin *in vitro* delayed apoptosis via JAK, NF-κB, and p38 MAPK signaling pathways, suggesting leptin as a survival cytokine (Wong et al., 2007), similar to neutrophils (Bruno et al., 2005). Furthermore, leptin also stimulated chemokinesis (Wong et al., 2007) and enhanced chemotactic migration of eosinophils isolated from human peripheral blood, in a dose-dependent manner, however, the underlying mechanisms remain unclear (Kato et al., 2011). In obese individuals, eosinophils demonstrated greater adhesion and chemotaxis toward eotaxin and RANTES (CCL5), compared with non-obese healthy volunteers (Grotta et al., 2013).

In human basophils, leptin treatment (10 nM) induced a strong migratory response, promoted the secretion of type 2 cytokines IL-4 and IL-13, and up-regulated the cell surface expression of CD63, which may have an exacerbating action on allergic inflammation (Suzukawa et al., 2011). Moreover, leptin is a survival-enhancing factor of human basophils, as aforementioned for eosinophils and neutrophils. Although leptin was a weak effect on direct induction of basophil degranulation, it potently primed basophils for enhanced degranulation in response to aggregation of IgE or its high-affinity receptor FcεRI (Suzukawa et al., 2011).

Altogether, these findings suggest leptin as a potent activator of neutrophils, eosinophils, and basophils through its positive action in cell survival, cytokines release and chemotaxis.

### Monocytes and Macrophages

Both isoforms of LEPR are expressed in PBMCs, being lower in cells from obese individuals compared with lean subjects (Tsiotra et al., 2000). Functional LEPR was also expressed in macrophages (O’Rourke et al., 2001). The effect of leptin on monocytes and macrophages has been well-established since its first evidence in Santos-Alvarez et al. (1999). Leptin promoted the proliferation of human circulating monocytes *in vitro* as well as its activation through induction of TNF-α and IL-6 production, and stimulation of surface markers, namely CD25, HLA-DR, CD38, CD71, CD11b, CD11c, and CD16 (Santos-Alvarez et al., 1999; Cannon et al., 2014). Moreover, leptin potentiated the stimulatory effect of LPS or PMA on human monocytes (Santos-Alvarez et al., 1999), and increased CCLs in cultured murine macrophages, being JAK2-STAT3 signaling pathway involved (Kiguchi et al., 2009). Leptin (625 nM) also augmented the production of several inflammatory mediators in monocytes/macrophages, such as interleukin 1 receptor antagonist (IL-1Ra) (Gabay et al., 2001), interferon-c-inducible protein (Meier et al., 2003), leukotrienes (Mancuso, 2004), nitric oxide (Dixit et al., 2003), and pro-inflammatory cytokines, namely TNF-α, IL-6, IL-1β, and resistin (Tsiotra et al., 2013; Scotece et al., 2014; Inzaugarat et al., 2017). By contrast, it was reported that 1 μg/ml leptin had no effect on IL-1β secretion but enhanced IL-18 in the THP-1 murine monocytic cell line. These apparent discrepancies could be species-specific (human vs. murine cells) and/or leptin treatment-dependent (1 μg/ml for 24 h vs. 1 μg/ml for 3 h). Additionally, recombinant leptin increased the expression of TLR2, but not TLR4, in human monocytes (Jaedicke et al., 2013).

A dose-dependent effect of leptin as a trophic factor to prevent apoptosis was found in serum-deprived human monocytes, being this effect mediated by the p42/p44 MAPK pathways (Najib and Sánchez-Margalet, 2002). Leptin (2 nM) stimulated the oxidative burst in monocytes (Sánchez-Pozo et al., 2003), and increased LPL expression through oxidative stress- and PKC-dependent pathways (Maingrette and Renier, 2003). Moreover, leptin promoted the phagocytosis of apoptotic cells by macrophages from lupus mice, via modulation of cAMP levels (Amarilyo et al., 2014). Leptin also promoted a defensive environment against *Leishmania donovani* infection by induction of macrophage phagocytic activity and intracellular ROS generation (Dayakar et al., 2016). Accordingly, macrophages from knocked-out *LepR* Tyr 985 mice presented reduced phagocytosis and killing activity of *Klebsiella pneumoniae* that was associated with diminished ROS production (Mancuso et al., 2012). It has been demonstrated that leptin-mediated protein radical formation, tyrosine nitration and activation of KCs are caused by peroxynitrite formation, which exacerbated NASH in diet-induced obese mice (Chatterjee et al., 2013). Concerning to chemoattractive activity, it was verified that leptin induced *in vitro* chemotactic responses for monocytes and macrophages (Curat et al., 2004; Gruen et al., 2007), via intracellular calcium influx, JAK/STAT, MAPK and PI3K pathways (Gruen et al., 2007). However, it has been reported that hematopoietic LEPR deficiency in mice did not change macrophage accumulation in WAT after diet-induced obesity versus wild-type mice (Gutierrez and Hasty, 2012). Likely, compensatory *in vivo* effects of other cytokines (like IL-1 or TNF-α) present in WAT could occur in obese individuals and which are absent in *in vitro* assays.

Leptin treatment (50 ng/ml) of human macrophages in culture, induced ‘alternatively activated’ or M2-phenotype surface markers, but they were able to secrete M1-typical cytokines (TNF-α, IL-6, IL-1β, IL-1ra, IL-10, MCP-1, and macrophage inflammatory protein 1-alpha (MIP-1α)), suggesting a role for leptin in the phenotype of macrophages found in adipose tissue (Acedo et al., 2013). In macrophages, leptin also triggered catecholamine-dependent increases in cAMP-histone deacetylase 4 signaling pathway, that reduced inflammation in adipose tissue (Luan et al., 2014). Additionally, leptin increased the expression of LEPR in M2 macrophages and stimulated IL-8 expression via p38 and ERK signaling pathways (Cao et al., 2016). In tumor-associated macrophages, leptin induced the expression of IL-18 via NF-kB, possible contributing to tumor progression (Li K. et al., 2016).

Macrophages are indirectly regulated by leptin through mast cells (Zhou et al., 2015). In particular, leptin expression was reduced in both human and mouse mast cells from lean adipose tissue compared with obese individuals. Leptin deficiency led to the anti-inflammatory activity of mast cells and, consequently, to a shift in macrophage polarization from M1 to M2; *in vitro* co-cultures of mast cells with BMDM increased IL-4-mediated arginase-1 and IL-10 expression, and suppressed LPS-mediated iNOS and IL-6 expression (Zhou et al., 2015). Furthermore, reduction of mast cells in leptin-deficient *ob/ob* mice exacerbated obesity and diabetes, indicating an important role of mast cells in obesity-related inflammation through its reactivity to leptin levels (Zhou et al., 2015).

Biologic drugs used for the treatment of psoriatic arthritis, namely adalimumab (an anti-TNF-α monoclonal antibody) and ustekinumab (a monoclonal antibody against the p40 subunit of IL-12 and IL-23), augmented LEPR expression in THP-1 human macrophages (Voloshyna et al., 2016). However, only ustekinumab was able to increase the expression of leptin, suggesting a novel mechanism for this biological drug. Further mechanistic studies focused on the leptin pathway could have potential therapeutic action in common obesity-related complications of psoriasis (Voloshyna et al., 2016). The establishment of *LepR*-deficient macrophage cell line DB-1, derived from differentiated bone marrow cells of *Lepr*-knockout mice, provide a powerful tool to study the role of leptin and its receptor in obesity-associated inflammation and immune system deregulation (Dib et al., 2016).

### NK Cells

The role of leptin in regulating NK cell development and activation was first verified in obese *Lepr*-deficient (*db/db*) mice, which showed decreased NK cell function (Tian et al., 2002). In this animal model, the population of NK cells in bone marrow was impaired through an increase in apoptotic rate, and recombinant leptin (200 ng/ml) significantly enhanced the survival of immature NK cells from wild-type mice via modulation of Bcl-2 and Bax gene expression (Lo et al., 2009). Furthermore, leptin administration (500 μg/kg) led to a higher activity of NK cells in lean animals (Nave et al., 2008). Consistently, human NK cells expressed functional long- and short-form of LEPR that influenced NK cell cytotoxicity through STAT3 activation and, consequently, transcription of genes encoding IL-2 and perforin (Zhao et al., 2003).

The above-mentioned results indicated that leptin signaling is required for normal NK cell immune function. However, there are some controversial findings concerning the time of leptin treatment *in vitro*. Short-term stimulation of human NK cells with leptin (50 nM) raised the secretion of IFNγ and cytotoxicity (Wrann et al., 2012; Laue et al., 2015). By contrast, long-term exposure to leptin decreased NK cell proliferation and immune function (Wrann et al., 2012). Obesity is partially characterized by a state of long-term, highly elevated leptin exposure, and NK cells from obese animals were significantly resistant to leptin stimulation (Nave et al., 2008), which could explain the functional desensitization of NK cells after long-term exposure. Accordingly, exposure of NK-92 human cell line to hyperleptinemia (similar to that observed in obese individuals) led to metabolic activation of NK-92 cells after 24 h, but there is a reduction of cell metabolism after 96 h (Lamas et al., 2013). Furthermore, obese individuals have lower NK function compared to lean individuals (Laue et al., 2015) and, after weight loss, the decrease of plasma leptin levels is accompanied by a restoration of IFNγ production by NK cell (Jahn et al., 2015; Bähr et al., 2017; Favreau et al., 2017).

Overall, leptin signaling seems to be necessary for normal NK cell immune function, increasing the immune activity and cell proliferation, and reducing the apoptotic rate of NK cells. Long-term exposure to hyperleptinemia, observed in obesity, has been associated with decreased NK immune activity possibly due to the development of leptin resistance. Further studies are needed to better understand the correlation between leptin levels and NK cell development and function, as well as the potential implications in obesity.

### Dendritic Cells

Human DCs, both immature and mature DCs, present functional active LEPR with the capacity to signal STAT-3 phosphorylation (Mattioli et al., 2009). Leptin (10 nM) acted as an activator of human DCs, evidenced by up-regulation of IL-1β, IL-6, IL-12, TNF-α and MIP-1α production, improvement of immature DCs migration (Mattioli et al., 2008; Al-Hassi et al., 2013) and their chemotactic responsiveness, licensing them toward Th1 priming (Mattioli et al., 2008). Moreover, leptin treatment promoted DC survival through decreased apoptosis via activation of NF-κB and PI3K-Akt signaling pathways, with a parallel increase of bcl-2 and bcl-x_L_ gene expression (Lam et al., 2006; Mattioli et al., 2009).

*Lepr*-deficient *db/db* mouse bone marrow culture displayed a reduced number of DCs, attributable to dysregulation of Bcl-2 genes and a consequent increase of apoptosis (Lam et al., 2006). Moreover, DCs from *db/db* mice possessed markedly reduced expression of co-stimulatory molecules and a Th 2-type cytokine profile, with a poor capacity to stimulate allogeneic T cell proliferation (Lam et al., 2006). Consistently, *db/db* DCs demonstrated down-regulation of PI3K/Akt and STAT-3 pathways (Lam et al., 2006). *Lep*-deficient *ob/ob* mice presented a reduced expression of DC maturation markers (CD40, CD80, and CD86), decreased production of inflammatory cytokines (IL-12, TNF-α, and IL-6), and augmented TGF-β production, but *ob/ob* mice-derived DCs were more efficient in inducing Treg or Th17 cells than wild-type animals (Moraes-Vieira et al., 2014). In DCs from *ob/ob* mice, leptin deficiency resulted in defective antigen presentation function toward *Leishmania donovani*, which was not reversed by leptin treatment (Maurya et al., 2016). Conversely, one report verified no changes in the phenotype, activation, antigen processing or presentation of DCs from leptin-knockout mice, but these cells showed an enhanced ability to activate T cells, suggesting that leptin may dampen T-cell responsiveness in the physiological context (Ramirez and Garza, 2014). Diet-induced obesity in mice fed with HFD results in an elevation of serum leptin levels and splenic CD11c^+^ DCs, with diminished DC cell stimulatory capacity, being these effects distinct from that caused by HFD alone in obese-resistant mice (Boi et al., 2016).

Altogether, these data demonstrated the important role of leptin in DC activation, chemoattraction, and survival, with possible implications in DC maturation and migration. Given the ability of DCs to orchestrate immune response and promote potent immunogenic responses through activation of T cell immunity, DCs-based immunotherapies to elicit immunity against cancer and infectious diseases are currently being developed. In particular, DCs can be differentiated *ex vivo*, exposed to antigens and induced to mature in the presence of adjuvants. Then, the mature DCs are injected into the patient and migrate to the lymph nodes to present antigens to T cells. Thus, the modulation of DCs maturation and activity by leptin is of most importance considering a potential application of leptin in immunotherapeutic approaches and as novel adjuvant immunopotentiator in vaccination protocols employing *ex vivo* generated autologous DCs.

## Leptin and Adaptive Immunity (**Figure 3**)

The role of leptin in adaptive immunity has first evidenced working with *ob/ob* and *db/db* mice, which showed thymus atrophy, T-cell lymphopenia, and impaired delayed-type hypersensibility (Lord et al., 1998; Howard et al., 1999; Matarese, 2000). Moreover, chronic leptin administration (1 μg/g body weight) reversed immunosuppressive status and thymic atrophy of *ob/ob* mice (Lord et al., 1998, *Nature*; Howard et al., 1999, *J. Clin. Inv.*). Since then, the role of leptin in T and B cell populations have been extensively studied.

### T Cells

T lymphocytes expressed the long form of LEPR (higher in peripheral CD4+ than in CD8+ T cells) (Lord et al., 1998; Kim et al., 2010), with signaling capacity to activate JAK-STAT pathway (Sanchez-Margalet and Martin-Romero, 2001). Consequently, leptin modulated cell proliferation, responsiveness, and polarization of T cells. Leptin dose-dependently promoted the proliferation of human naïve (CD45RA+) CD4+ T cells, whereas it minimally affected memory (CD45RO+) CD4+ T cells proliferation (Lord et al., 1998, 2002). Additionally, morbidly obese children, who were congenitally deficient in leptin, presented a decreased number of circulating CD4+ T cells, as well as impaired T cell proliferation and cytokine release, which were reversed by administration of recombinant human leptin (Farooqi et al., 2002). Moreover, leptin inhibited autophagy in human CD4+CD25- conventional T cells via mTOR pathway (Cassano et al., 2014), which emerged as the potential link between immunity and nutritional status (Procaccini et al., 2012).

#### T Helper Cells

Leptin also promoted CD4+ T cell polarization toward a Th1 response (which secretes IFNγ and IL-2) rather than Th2 response (which secretes IL-4) (Martín-Romero et al., 2000). Accordingly, under Th2-polarizing conditions, the *in vitro* leptin treatment decreased IL-4-producing T cells and inhibited T cell proliferation (Batra et al., 2010). However, it was recently reported that *in vivo* leptin-deficiency attenuated allergic airway inflammation and that high leptin levels associated with obesity promoted proliferation and survival of Th2 lymphocytes, as well as the production of type 2 cytokines, altogether contributing to allergic responses (Zheng et al., 2016). Besides that, leptin was involved in thymus morphology and functions (Lamas et al., 2016), particularly in thymocyte differentiation of double positive CD4+CD8+ T cells into single positive CD4+ T cells (Kim et al., 2010).

IL-17-producing Th cells (Th17) have a crucial role in the promotion and maintenance of inflammatory and autoimmune pathologies. Leptin was demonstrated to increase Th17 population and responsiveness in SLE, via retinoic acid-related orphan receptor (ROR)γt (Yu et al., 2013; Fujita et al., 2014; Reis et al., 2015). In collagen-induced arthritis mouse model, articular injection of leptin (5 μg) increased the number of Th17 in the joint tissue, resulting in exacerbating joint inflammation, and consequently early onset of arthritis and increased disease severity (Deng et al., 2012). Leptin, in concentrations similar to that found in blood during pregnancy, promoted the differentiation of peripheral blood CD4+ cells to Th17 cells, but suppressed the formation of Treg cells *in vitro* (Orlova and Shirshev, 2014). CD4+ T cell-derived leptin, but not plasma leptin, were positively correlated with the percentage of Th17 cells or RORγt levels in chronic lymphocytic thyroiditis, an organ-specific autoimmune disease (Wang et al., 2013). Furthermore, *Lepr*-deficient CD4+ T cells verified a reduced capacity for Th17 differentiation, via down-regulation of STAT3 activation (Reis et al., 2015).

#### T Regulatory Cells (Treg)

Leptin also regulated CD4+CD25+ Treg proliferation (Matarese et al., 2010). Treg lymphocytes play a critical role in controlling the inappropriate immune responses characteristic of autoimmune diseases and allergy. In humans, leptin negatively affected the proliferation of Foxp3+CD4+CD25+ Treg; *in vitro* leptin neutralization, during anti-CD3 and anti-CD28 stimulation, led to the proliferation of the isolated human Treg cell (De Rosa et al., 2007). Obese individuals presented a reduced number of CD4+CD25+CD127-Foxp3+ Treg cells, which was correlated with body weight, BMI, and plasma leptin levels (Wagner et al., 2013). Moreover, leptin-deficient mice presented an increased percentage of peripheral Treg, compared with wild-type mice, which is reversed after leptin administration (De Rosa et al., 2007). It was verified that leptin played an important role in Treg dysfunction in patients with pulmonary arterial hypertension (Huertas et al., 2016). Accordingly, *Lepr*-deficient rats developed less severe hypoxia-induced pulmonary hypertension and were protected against decreased Treg function after exposure to hypoxia (Huertas et al., 2016). In SLE, the disease-associated higher leptin serum levels were negatively correlated with disease severity and number of Treg cells (Ma et al., 2015; Margiotta et al., 2016; Wang et al., 2017), and fasting-induced hypoleptinaemia was related to Treg population recovery in lupus-prone mice (Liu et al., 2012). Leptin-deficient *ob/ob* mice and a mouse model of lupus with leptin deficiency demonstrated increased frequency of Tregs cells (Fujita et al., 2014; Lourenço et al., 2016). These data evidenced the potential of anti-leptin-based approaches for immune system-dysregulated pathologies associated with reduced Treg function, such as SLE, obesity, T2DM, and metabolic syndrome.

T cell metabolism is directly related to its function (MacIver et al., 2013); effector T cells, such as Th1 and Th17, demand a high glycolytic metabolism to fuel proliferation and function, while Treg cells require oxidative metabolism to fuel suppressive activity. Recently, leptin was found to directly promote T-cell glycolytic metabolism and consequently induce Th17 cell differentiation, being Treg cells unchanged, in a mouse model of experimental autoimmune encephalomyelitis (Gerriets et al., 2016). Leptin regulated glucose metabolism partly by up-regulation of glucose transporter Glut1 (Saucillo et al., 2014). Moreover, fasting led to decreased ability of T cells to secrete IL-2 and IFNγ, and inability to up-regulate glucose uptake and glycolytic flux (Saucillo et al., 2014), while Treg expansion was increased (Liu et al., 2012); leptin administration (1 μg/g body weight) rescued peripheral T cell function and metabolism in fasted mice (Saucillo et al., 2014). Likely, fasting was extensively reported to be associated with immune deficiency and increased susceptibility to infection (Gerriets and MacIver, 2014). Thus, leptin seems to provide a key link between nutritional status and inflammatory T cell responses (Gerriets et al., 2016; De Rosa et al., 2017).

Altogether, these data revealed the ability of leptin to increase immune activity by modulation of T cell number and function. Leptin can promote proliferation of naive T cells, as well as Th1 and Th17 proliferation and cytokine production. Moreover, leptin decreases Treg cell proliferation. Considering the regulatory effects of leptin on Th17 and Treg populations, revoking leptin signaling might be a potential therapeutic approach for inflammation and autoimmunity.

### B Cells

In contrast to macrophages and T cells, little is known about the role of leptin in the B lymphocytes development and function. B cells expressed the long form of the LEPR, suggesting a direct effect of leptin on B cell function (Busso et al., 2002). Accordingly, *db/db* and *ob/ob* presented a reduced number of peripheral blood and bone marrow B lymphocytes, which was recovered after leptin treatment (Bennett et al., 1996; Claycombe et al., 2008). Conversely, *db/db* mice presented an increased absolute number of B cells in the peritoneal cavity (Jennbacken et al., 2013), and the increase of leptin was correlated with a decrease in B cells of mice with unbalanced diets (carbohydrate-rich and fat-rich) (Martínez-Carrillo et al., 2015). Thus, further investigation is needed to better clarify the role of leptin in lymphopoiesis. Leptin promoted B cell homeostasis through inhibition of apoptosis and induction of cell cycle entry via Bcl-2 and cyclin D1 activation (Lam et al., 2010). Furthermore, leptin dose-dependently activated human peripheral blood B cells, inducing the secretion of pro-inflammatory cytokines, namely TNF-α and IL-6, and the anti-inflammatory cytokine IL-10, via JAK-STAT and p38MAPK-ERK1/2 signaling pathways (Agrawal et al., 2011). Likewise, leptin (50 ng/ml) activated and induced the production of higher amounts of TNF-α, IL-6, and IL-10 by B cells from aged subjects compared to young individuals (Gupta et al., 2013), which is associated with leptin-mediated STAT3 phosphorylation (Gupta et al., 2013; Frasca et al., 2016).

Leptin can also modulate B cell development – decrease pro-B, pre-B and immature B cells and increase mature B cells- in bone marrow of fasted mice, characterized by low serum leptin levels (Tanaka et al., 2011). Leptin administration reversed the starvation-induced lymphopenia of bone marrow B cells, indicating an important role of central leptin in the immune system (Tanaka et al., 2011; Fujita et al., 2012). Moreover, leptin might regulate B cell activity in obesity (Nikolajczyk, 2010; Frasca et al., 2016). In particular, B cells were described to accumulate in murine VAT and to critically regulate T2DM-associated inflammation through activation of CD8+ and Th1 cells and release of pathogenic antibodies (Winer et al., 2011; DeFuria et al., 2013).

In summary, leptin can increase B cell population by augmenting proliferation and reducing the apoptotic rate, activate B cell to secrete pro-, anti- and regulatory cytokines, and also modulate B cell development.

## Leptin and Immune-Metabolic Pathologies

### Leptin and Obesity-Associated Metabolic Disorders

Obesity is associated with life-threatening co-morbidities, including insulin resistance, T2DM, NAFLD and steatohepatitis (NASH) (Lebovitz, 2003; Kamada et al., 2008; Klöting and Blüher, 2014; Fasshauer and Blüher, 2015). Adipokines, in particular leptin, mediate the crosstalk between adipose tissue and metabolic organs (especially liver, muscle, pancreas and central nervous system) (Cao, 2014). Thus, leptin has emerged as a significant pathological component in the development of metabolic disorders (DePaoli, 2014).

#### Type 2 Diabetes Mellitus

T2DM is the most significant obesity-associated metabolic disorder and their prevalence is increasing worldwide in parallel (Bhupathiraju and Hu, 2016). Leptin has been proposed as a therapeutic target of T2DM, for its impact on food intake and body weight as well as its potential to improve insulin action (Kalra, 2009). Interestingly, leptin-deficient mice (Pelleymounter et al., 1995) and human (Farooqi et al., 1999, 2002; Ozata et al., 1999) have diabetic features, which were reversed with leptin replacement. The anti-diabetic effect of leptin is mediated by activation of IRS-PI3K pathway that improved insulin sensitivity in peripheral tissues (Morton et al., 2005). Activation of JAK2/IRS/PI3K/Akt signaling pathway by leptin and insulin triggers the translocation of glucose transporter type 4 (GLUT4) from cytosol to cell surface, and glucose uptake (Benomar et al., 2006; Zhao and Keating, 2007). Moreover, in the liver, leptin deficiencies decrease AMPK activity (Namkoong et al., 2005), which is also involved in glucose homeostasis regulation (Schultze et al., 2012). Leptin has also been implicated in the regulation of insulin secretion by pancreatic β-cells (Kulkarni et al., 1997), as well as in peripheral insulin resistance (Silha et al., 2003; Yadav et al., 2011). However, clinical trials to evaluate the potential of leptin monotherapy in obese humans with T2DM failed to demonstrate therapeutic activity acutely or chronically, with no observation of important weight loss or metabolic improvements (insulin sensitization, amelioration of glucose and lipid metabolism) (Mittendorfer et al., 2011; Moon et al., 2011; Wolsk et al., 2011). In this context, unresponsiveness to leptin – leptin resistance, caused by hyperleptinemia observed in obese humans, should be considered (Frederich et al., 1995). Further understanding of leptin resistance mechanisms could enable new leptin targeted therapies for obesity and diabetes in specific subsets of patients. In fact, leptin therapy improved the diabetic condition in children (Farooqi et al., 1999, 2002) and adults (Licinio et al., 2004) with familial leptin deficiency, and in lipoatrophic diabetes (Oral et al., 2002).

Importantly, T2DM is associated with altered components of immune system, including modified levels of specific chemokines and cytokines, changed number and activation status of leukocytes populations and increased apoptosis and tissue fibrosis (Donath, 2014), all promoted by obesity-associated inflammation in adipose tissue (Donath and Shoelson, 2011). Given the modulator action of leptin in innate and adaptive immune system (deeply described above), it is rational to see leptin as a linker of T2DM development, not only with metabolism but also with inflammation. Indeed, in patients with newly diagnosed T2DM, leptin levels were correlated with CRP, an inflammatory marker broadly evaluated for its association with risk factors for T2DM pathology (Morteza et al., 2013).

#### Non-alcoholic Fatty Liver Disease

NAFLD, the major cause of chronic liver illness in developed countries, comprises a wide group of pathologies primary caused by a buildup of fat in the liver that spans from simple steatosis to NASH, liver fibrosis, cirrhosis, and hepatocellular carcinoma (Tiniakos et al., 2010). Given that NAFLD is increasing worldwide and it is associated with high-incident extra-hepatic complications such as obesity, T2DM, cardiovascular diseases and chronic kidney disease (Byrne and Targher, 2015; Polyzos et al., 2015), great efforts have been made in the last years to unravel the mechanisms underlying the disease pathophysiology and further development of effective NAFLD therapies.

Hepatic inflammation and hepatocyte injury and death are hallmarks of NAFLD/NASH. Fat overload by hepatocytes causes lipotoxicity and the release of DAMPs which activated Kupfer cells (KC; specialized liver macrophages) and HSC promoting inflammation and fibrosis, respectively. KC activation plays a central role in NAFLD pathophysiology through the production of pro-inflammatory cytokines and chemokines such as TNF-α, IL-1β, IL-6, CCL2 and CCL5, that contributed to leukocyte infiltration and inflammatory necrosis of hepatocytes, and fibrogenesis (Arrese et al., 2016). Dysbiosis of the gut microbiota may also conduct to KC activation through PAMPs, which originate in the gut and reach liver via portal circulation due to altered intestinal permeability. Other immune cells have been implicated in the NAFLD pathophysiology, although its role is less clear (Arrese et al., 2016). NK cells were impaired in experimental NASH, while natural killer T cells (unique immune cell subtype that expresses NK cells surface markers as well as T-cell antigen receptor) are depleted in steatosis but increased later during disease progression likely leading to inflammation and fibrosis in NASH via the production of IL-4, osteopontin, and IFN-γ (Tajiri and Shimizu, 2012; Tian et al., 2013). Neutrophils exacerbate the ongoing inflammation through macrophage recruitment and cell damage via the release of myeloperoxidase, ROS, and elastase (Xu R. et al., 2014). The role of DCs in NASH is complex and somehow controversial. DCs rapidly infiltrate into the liver in experimental NASH exhibiting an activated immune phenotype, but its depletion exacerbates hepatic inflammation (Tacke and Yoneyama, 2013; Arrese et al., 2016). B- and T-cells also contributed to hepatic inflammation via secretion of pro-inflammatory cytokines that stimulated KC activation (Arrese et al., 2016).

Considering the strong metabolic and inflammatory components of NAFLD, leptin is regarded as a key regulator of NAFLD physiopathology (Polyzos et al., 2015). Leptin seems to feature a dual activity in NAFLD experimental models by exerting an early protective anti-steatosis effect in the initial stages of the disease, and a late pro-inflammatory and pro-fibrogenic action, when the disease persists or progress (Polyzos et al., 2015). In leptin-resistant Zucker fa/fa diabetic fatty rats, the expression of SREBP-1c (master regulator of glucose metabolism, and lipid and fatty acid production) is increased in liver (Kakuma et al., 2000), and infusion of adenovirus-leptin decreased hepatic triglyceride synthesis and β-oxidation via SREBP-1c down-regulation and PPARα up-regulation (Lee et al., 2002), thus preventing hepatic lipid accumulation.

Through anti-steatotic effect, leptin can ultimately lead to hepatic detrimental effects. Leptin activated HSCs, leading to up-regulation of pro-inflammatory and pro-angiogenic factors expression (like angiopoietin-1 and vascular endothelial growth factor), as well as collagen α1 and tissue inhibitor of metalloproteinase-1, ultimately acting as hepatic fibrogenesis inducer (Polyzos et al., 2015). Activated HSCs were able to secrete leptin, thus establishing a vicious cycle that further promotes liver fibrosis (Polyzos et al., 2015). Moreover, leptin was reported as potent HSCs mitogen and to prevent HSCs apoptosis, hence promoting the pathogenesis of hepatic fibrosis. Leptin (200 nM) increased the expression of TGF-β1 in KCs and sinusoidal endothelial cells, and connective tissue growth factor in KCs (Ikejima et al., 2002), being KCs-HSCs cross-talk proposed for liver fibrosis (Wang et al., 2009). Additionally, leptin is involved in macrophage-mediated KCs activation via induction of oxidative stress in macrophages (Chatterjee et al., 2013). Compared to healthy subjects, NAFLD patients demonstrated an increased leptin-stimulated TNFα and ROS production in peripheral monocytes, as well as IFNγ production in circulating CD4+ cells (a marker of Th1 differentiation) (Inzaugarat et al., 2017). Altogether, these data elucidated the role of leptin in NAFLD by modulation of HSCs, KCs, and inflammatory cells response.

In *ob/ob* mice, the congenital absence of leptin abrogated the development of CCl_4_-induced hepatic fibrosis comparing to lean littermates, which is reverted by leptin treatment (100 ng/ml) (Saxena et al., 2002). Likewise, xenobiotics- or thioacetamide-induced hepatic fibrosis was prevented in Zucker fa/fa rats, being involved the activation of HSCs and expression of procollagen-I and TGF-β1 (Ikejima et al., 2005). In humans, the role of leptin is controversial. Although leptin serum levels were initially related with hepatic steatosis but not with necroinflammation or fibrosis (Chitturi et al., 2002), later studies failed to demonstrate any significant association (Tsochatzis et al., 2009). Recombinant leptin has been successfully used in the treatment of insulin resistance and hepatic steatosis in patients with lipodystrophy and NASH (Oral et al., 2002; Petersen et al., 2002; Javor et al., 2005). However, large-scale and well-designed prospective cohort studies are necessary to deeply elucidate the role of leptin in hepatic lipid handling, inflammation, and fibrosis along with the identification of NAFLD patients subsets that may benefit from therapies directed to leptin system.

### Leptin in Rheumatic Diseases

**FIGURE 4:**
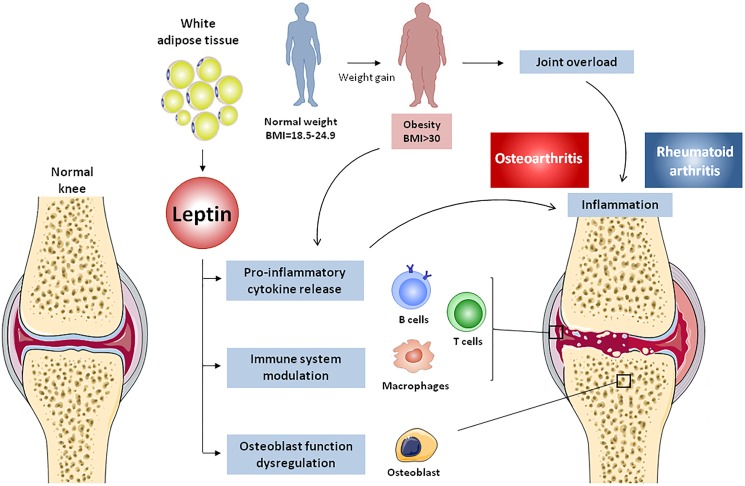
Effects of adipose tissue-derived leptin on osteoarthritis and rheumatoid arthritis. Body weight gain, accompanied by white adipose tissue expansion, lead to obesity and subsequent increase of mechanical load, resulting in cartilage degradation and osteoarthritis onset. Adipose tissue-derived leptin causes osteoblast dysregulation in subchondral bone, thus promoting joint destruction. Additionally, leptin induces pro-inflammatory cytokine release from innate and adaptive immune cells, generating an inflammatory environment that prompts cartilage damage and rheumatoid arthritis.

Leptin has been described as a key factor in the pathophysiology of rheumatic diseases due to its capability to modulate bone and cartilage metabolism and to influence innate and adaptive immune responses (**Figure 4**).

#### Osteoarthritis

Osteoarthritis, the most common joint disease, is a painful and debilitating illness characterized by progressive degeneration of articular joints. Initially seen as simply “wear and tear” disease, OA is currently considered a complex and multifactorial pathology triggered by inflammatory and metabolic imbalances that affect the entire joint structure (articular cartilage, meniscus, ligaments, bone, and synovium) (Loeser et al., 2012). Leptin levels are increased in serum, infrapatellar fat pad (IPFP), synovial tissues, and cartilage of OA patients compared to healthy individuals (Dumond et al., 2003; de Boer et al., 2012; Conde et al., 2013). Accordingly, leptin-deficient or *LepR*-deficient mice developed extreme obese phenotype without increased incidence of knee OA, suggesting that leptin signaling is essential to the development and progression of obesity-associated OA (Griffin et al., 2009). Furthermore, long form of LEPR was found to be expressed in human cartilage cells – chondrocytes (Figenschau et al., 2001).

Some initial findings suggested an anabolic role of leptin in cartilage. In particular, exogenous leptin administration (30 μg) stimulated proteoglycan and growth factors (insulin-like growth factor-1 and TGF-β) synthesis in rat knee-joint cartilage (Dumond et al., 2003). However, most of the studies reported a catabolic role of leptin underlying OA pathogenesis. A recent study determining the gene expression profile of leptin-induced articular rat cartilage by microarray analysis, associated the up-regulation of matrix metalloproteinases (MMPs), inflammatory factors, growth factors and osteogenic genes with leptin-induced OA phenotype (Fan et al., 2018). Our group demonstrated that leptin (400 or 800 nM), in synergy with IFNγ or IL-1β, activated type 2 nitric oxide synthase (NOS2) via JAK2, PI3K and MAPK (MEK1 and p38) pathways, in cultured human and murine chondrocytes (Otero et al., 2003, 2005, 2007). Nitric oxide (NO), is a well-known pro-inflammatory mediator which lead to joint degradation through induction of chondrocyte phenotype loss, apoptosis and metalloproteinase (MMP) activity (Rahmati et al., 2016). Leptin (800 nM), alone or in combination with IL-1β, also induced the expression of COX-2 and the production of PGE2, IL-6, and IL-8 in cartilage explants of OA patients and human primary chondrocytes (Vuolteenaho et al., 2009; Gomez et al., 2011), revealing that leptin contributed to the pro-inflammatory environment of OA cartilage. It was also demonstrated that leptin (500 ng/ml) enhanced IL-6 production, mediated by chondrocyte-synovial fibroblast cross-talk, in OA patients (Pearson et al., 2017). Moreover, leptin modulated the production of inflammatory mediators by immune cells. In particular, the production of IL-6, IL-8, and CCL3 were increased by leptin in CD4+ T cells from OA patients, but not from healthy subjects (Scotece et al., 2017); thus, demonstrating new insights into the role of leptin in the immune system and OA pathophysiology.

Leptin can directly induce the expression of MMPs that are involved in OA-related joint destruction, like MMP-1 (also known as interstitial collagenase), MMP-3 (also known as stromelysin), and MMP-13 (also known as collagenase), via NF-κB, PKC, and MAPK pathways (Bao et al., 2010; Koskinen et al., 2011; Hui et al., 2012). MMP-2 (72 kDa type IV collagenase), MMP-9, and disintegrin and metalloproteinase with thrombospondin motifs (ADAMTS) 4 and ADAMTS5, were also increased by leptin, while fibroblast growth factor 2 and proteoglycan were down-regulated (Bao et al., 2010; Conde et al., 2011b). Leptin (800 nM) can perpetuate the cartilage-degradation processes due to induction of VCAM-1, an adhesion molecule responsible for leukocyte and monocyte chemotaxis and infiltration to inflamed joints, via JAK2 and PI3K pathways in chondrocytes (Conde et al., 2012; Vestweber, 2015). SOCS-3 was pointed as a regulator of leptin-induced expression of MMP-1, -3, and -13, and pro-inflammatory mediators IL-6, NO and COX-2 (Koskinen-kolasa et al., 2016). Additionally, leptin increased the production of other adipokines, namely lipocalin-2, by human cultured chondrocytes (Conde et al., 2011a).

MicroRNAs, small single-stranded non-coding segments of RNA, are increasingly recognized as regulatory molecules involved in disease processes, including OA, inflammation, and obesity (Marques-Rocha et al., 2015; Deiuliis, 2016; Nugent, 2016). miR-27 was found to be decreased in OA chondrocytes and to directly targeted the 3′-untranslated region of leptin (Zhou et al., 2017). Furthermore, the injection of OA rats with miR-27 lentiviral overexpression vector resulted in decreased levels of IL-6 and -8, as well as MMP-9 and -13, thus indicating the protective action of miR-27 in OA, possibly by targeting leptin.

Chondrogenic progenitor cells as cartilage seed cells are crucial to maintain cartilage homeostasis and replace damaged tissue (Seol et al., 2012). Leptin (50 ng/ml) can reduce CPCs migratory ability and their chondrogenic potential, and augment CPCs osteogenic transformation, hence changing CPC differentiation fate (Zhao et al., 2016). CPC cell cycle arrest and senescence are also induced by leptin (Zhao et al., 2016). Furthermore, leptin influenced the regulation of bone metabolism through induction of abnormal osteoblast function, which is associated to joint destruction in OA patients (Findlay and Atkins, 2014; Conde et al., 2015). The augmented production of leptin by OA subchondral osteoblasts is related with *in vitro* elevated levels of alkaline phosphatase, osteocalcin, collagen type 1, and TGF-β1, all being responsible for dysregulated osteoblast function (Mutabaruka et al., 2010). Additionally, bone morphogenic protein (BMP)-2 is increased in leptin-stimulated human primary chondrocytes (Chang et al., 2015). Leptin also suppressed bone formation *in vivo* (Ducy et al., 2000), but there are some discrepancies with *in vitro* results.

Taking together, this evidence indicated a key role of leptin in OA pathophysiology by influencing pro-inflammatory status, cartilage catabolic activity, as well as cartilage and bone remodeling. However, studies in a large cohort of patients are needed to better clarify the leptin significance in the development and progression of OA.

#### Rheumatoid Arthritis

Rheumatoid arthritis is a chronic inflammatory joint illness characterized by synovial membrane inflammation and hyperplasia (“swelling”), production of autoantibodies, namely rheumatoid factor and anti-citrullinated protein antibody – autoimmune disease, destruction of cartilage and bone (“deformity”), and systemic features including skeletal, cardiovascular, pulmonary, and psychological complications (McInnes, 2011; Smolen et al., 2016). Evidencing the crucial role of immune system in RA pathology, RA-associated synovitis comprises both innate immune cells (like monocytes, DCs, and mast cells) and adaptive immune cells (like Th1, Th17, and B cells) (McInnes, 2011; Smolen et al., 2016). As described above, leptin modulates neutrophils chemotaxis, activates proliferation and phagocytosis of monocytes and/or macrophages, regulates NK cytotoxicity, induces proliferation of naïve T cells, promotes Th1 cell immune response and down-regulates Th2 cell immune response. Moreover, leptin modulates the activity of Treg cells, which are potent inhibitors of autoimmunity, thus having a potential implication in RA pathophysiology (Toussirot et al., 2015).

Several studies have found a positive correlation between serum and synovial leptin levels and RA pathology (Otero et al., 2006; Targońska-Stępniak et al., 2008; Yoshino et al., 2011; Olama et al., 2012), but there are controversial results (Anders et al., 1999; Popa et al., 2005; Hizmetli et al., 2007; Oner et al., 2015). Differing results may be due to the relatively small sample size, inconsistency of the baseline characteristics of participants (age, race, disease duration, BMI, …), co-existence of other auto-immune diseases, employment of different methods to measure leptin levels in RA patients, or underlying patients treatments that intervene with the endocrine system. The present consensus is that leptin levels are elevated in RA patients, and serum and synovial fluid levels of leptin were associated with disease duration and parameters of RA activity (Olama et al., 2012; Lee and Bae, 2016), although large cohorts studies are necessary. Experimental animal models of arthritis had demonstrated the leptin action in joint inflammation. In particular, compared with control mice, leptin-deficient mice presented a less severe antigen-induced arthritis, decreased levels of TNF-α and IL-1β in knees synovium, and an impaired antigen-specific T cell proliferative response with lower IFN-γ and higher IL-10 production, which indicates a shift toward Th2 cell response (Busso et al., 2002). Accordingly, injection of leptin (5 μg) into the knee joint of collagen-immunized mice augmented arthritis severity, accompanied by elevated synovial hyperplasia and joint damage through enhancement of Th17 cell response (Deng et al., 2012). In fact, clinical trials using a humanized anti-IL-17 monoclonal antibody added to oral disease-modifying anti-rheumatic drugs, demonstrated improved signs and symptoms of RA, indicating the therapeutic potential of IL-17-directed strategies (Genovese et al., 2010).

Since leptin modulates the immune system, as well as insulin resistance and metabolic disorders like metabolic syndrome and obesity, all RA-associated conditions, this adipokine represents an attractive therapeutic target for RA. Accordingly, reducing leptin levels in RA patients by fasting improved the clinical symptoms of the disease (Fraser et al., 1999). Amidst the possible therapeutic approaches to antagonize leptin actions in RA, are leptin mutants with antagonist activity, and monoclonal antibodies against human LEPR or leptin itself (Tian et al., 2014). Interestingly, clinical studies evaluating the effect of drug modulators of insulin sensitivity (affected by leptin levels as described above), such as PPARγ agonists, are ongoing to provide new potential treatment to improve the inflammatory status and cardiovascular outcome in RA patients (Chimenti et al., 2015). Further understanding of leptin mechanisms would be of utmost importance for RA treatment.

Hence, leptin can be pointed as a link between immune tolerance, metabolic function, and autoimmunity, and leptin signaling-directed strategies could provide future innovative therapies for autoimmune disorders like RA.

#### Systemic Lupus Erythematosus

Systemic lupus erythematosus is a chronic autoimmune disorder of unclear etiology characterized by hyperactive T and B cells, autoantibody production, deposition of immune complex, elevated blood levels of pro-inflammatory cytokines and multisystem organ damage, encompassing from mild manifestations (non-erosive arthritis or skin rash) to life-threatening complications (lupus nephritis, neuropsychiatric disorders, cardiovascular disorders, and metabolic syndrome). Although the pathogenesis of SLE is poorly understood, genetic, hormonal and environmental factors have been implicated in the onset of this heterogeneous disease, which predominantly affects women of childbearing age (Gatto et al., 2013; Liu and La Cava, 2014).

Several studies suggest the implication of adipokines, namely leptin, in the pathogenesis of SLE. Although some reports found no statistical association between disease activity and leptin levels (Li H.M. et al., 2016), recently, a meta-analysis of eighteen studies determined that serum/plasma leptin levels were significantly elevated in SLE patients (Lee and Song, 2018). Furthermore, leptin has been suggested as a player that affects the cardiovascular risk in SLE patients. Accordingly, leptin and HFD induced proinflammatory high-density lipoproteins and atherosclerosis in BWF1 lupus-prone mice, and leptin levels were correlated with BMI, disease activity index (SLEDAI), as well as insulin and CRP levels, all CVD risk factors, in SLE patients (Xu W.D. et al., 2014).

The role of leptin in SLE development has been investigated using leptin-deficient (ob/ob) mice treated with lupus-inducing agent (Lourenço et al., 2016). Leptin deficiency protected mice from the development of autoantibodies as well as renal disease, and elevated the levels of Treg cells, compared with wild-type controls. Moreover, in (NZBxNZW)F1 lupus-prone mice, leptin administration accelerated the development of autoantibodies and renal disease, while leptin antagonism delayed disease progression (Lourenço et al., 2016). At cellular level, leptin promoted Th1 responses in human CD4+ T cells and in lupus-prone mice via RORγ transcription, whereas leptin neutralization inhibited Th17 responses in autoimmune-prone mice (Yu et al., 2013). Additionally, fasting induced hypoleptinemia or leptin-deficient mice demonstrated decreased levels of Th17 and elevated levels of Treg cells (Liu et al., 2012). In SLE, apoptotic cells represent the major source of self antigens that promote and fuel autoimmune responses. Leptin promoted T cell survival and proliferation of autoreactive T cells in mice with an autoreactive T cell repertoire, including (NZBxNZW)F1 lupus-prone mice (Amarilyo et al., 2013). Leptin also promoted phagocytosis of apoptotic cells by macrophages in lupus-prone mice, which increase the availability of apoptotic-derived antigens to T cells and subsequent development of self-antigen-reactive T cells (Amarilyo et al., 2014).

Altogether, these data support the involvement of leptin in the development of SLE. However, further investigations are needed to fully understand the role of leptin in SLE and thus, explore this adipokine as potential therapeutic target of SLE.

## Conclusion and Future Outlook

Obesity and its comorbidities, such as T2DM, non-alcoholic fatty acid liver disease, OA and RA, reached epidemic proportions and are still rising in developing countries. Anti-obesity therapeutic options have provided only a limited long-term efficacy (lifestyle changes, physical activity, diet, and pharmacotherapies) or are not completely safe (bariatric surgery) (Zhang et al., 2014). Therefore, it has become increasingly relevant to disclose new clinical biomarkers and to develop innovative therapeutic strategies for obesity-associated pathologies and chronic inflammation.

The adipose tissue-derived factor leptin has been emerged as a key regulator of nutritional state and metabolism, as well as a modulator of immune system activation and innate-adaptive frontier; thus bridging obesity with metabolic disorders (T2DM and NAFLD) and inflammatory pathologies that affect bones and joints (OA and RA). Consequently, plasma leptin concentration could be a biological marker of the inflammatory status and the onset and evolution of pathologies associated with dysregulation of the immune system, and hereafter evaluations will be essential to establish leptin as clinical biomarker. Moreover, control of bioactive leptin levels by high-affinity leptin-binding molecules, miRNAs targeting leptin, LEPRs antagonists or monoclonal humanized antibodies against LEPR are likely to be feasible therapeutic approaches (Otvos et al., 2011). Recombinant leptin is already available for use in patients with leptin congenital deficiency while the synthetic leptin analog metreleptin has been approved for lipodystrophy treatment (Tchang et al., 2015). Importantly, the development of antibodies that could cross-react with endogenous leptin and cause an effective leptin-deficient state responsible for the loss of efficacy and infection has become a significant concern (DePaoli, 2014). However, given the pleiotropic action of leptin, a systematic approach to modulate their levels and thus prevent obesity-associated disorders might be, for the moment, unavailable. Instead, strategies targeting leptin’s actions precisely and in specific immune cell subpopulations, or targeting of specific receptor isoforms, could be a potential viable option to add novel therapeutic agents against immune-metabolic pathologies. It is now clear that leptin is an important regulator of metabolic status and influence inflammatory and immune responses in several diseases. Nonetheless, leptin network is complex and a lack of a full understanding of leptin’s immunomodulatory mechanisms in almost all of the cells of immune system and its potential side effects are still problems that need to be figured out in drug discovery. Further insights into the pathophysiological role of leptin in the immune system and in obesity-associated disorders will be of great importance for the development of novel therapeutic approaches for these diseases.

## Author Contributions

VF and JP have made a substantial contribution to acquisition and analysis of data and critically revised it. VC-C, CR-F, AM, MG-G, and RG have been involved in drafting the manuscript and revising it critically for important intellectual content. OG made a substantial contribution to conception and design of the review article, drafting the manuscript, and critically revising it. All authors approved the final version to be published.

## Conflict of Interest Statement

The authors declare that the research was conducted in the absence of any commercial or financial relationships that could be construed as a potential conflict of interest.

## Abbreviations

ADAMTS: a disintegrin and metalloproteinase with thrombospondin motifs
AMPK: adenosine monophosphate-activated protein kinase
Arg-1: arginase-1
BMDM: bone marrow-derived macrophages
BMI: body mass index
cAMP: cyclic adenosine monophosphate
CCL: CC-chemokine ligand
CD: cluster of differentiation
COX-2: cyclooxygenase-2
CPCs: chondrogenic progenitor cells
CRP: C-reactive protein
DAMPs: damage-associated molecular patterns
DCs: dendritic cells
ERK: extracellular signal-regulated kinase
GLUT: glucose transporter
HFD: high-fat diet
HLA-DR: human leukocyte antigen-antigen D related
HSC: hepatic stellate cell
ICAM: intercellular adhesion molecule
IFNγ: interferon γ
IL: interleukin
iNOS: inducible nitric oxide synthase
IPFP: intrapatellar fat pad
IRS: insulin receptor substrate
JAK: Janus kinase
JNK: c-jun N-terminal kinase
KC: kupffer cells
LEPR: leptin receptor
LPL: lipoprotein lipase
LPS: lipopolysaccharide
MAPK: mitogen-activated protein kinase
MCP-1: monocyte chemoattractant protein 1
MEK: mitogen-activated protein kinase kinase
MIP-1α: macrophage inflammatory protein-1 alpha
miRNA: microRNA
MMPs: matrix metalloproteinases
NAFLD: non-alcoholic liver disease
NASH: non-alcoholic steatohepatitis
NF-κB: nuclear factor- κB
NO: nitric oxide
NOS2: nitric oxide synthase 2
OA: osteoarthritis
PAMPs: pathogen-associated molecular patterns
PGE2: prostaglandin E2
PI3K: phosphoinositide 3-kinase
PKC: protein kinase C
PMA: phorbol myristate acetate
PMNs: human polymorphonuclear neutrophils
PPARα: peroxisome proliferator-activated receptor alpha
RA: rheumatoid arthritis
RORγt: retinoic acid-related orphan receptor gamma t
ROS: reactive oxygen species
SLE: systemic lupus erythematosus
SREBP-1c: sterol regulatory element-binding protein-1c
STAT: signal transducer and activator of transcription
T2DM: type 2 diabetes mellitus
TGF-β: transforming growth factor
Th: T helper cells
TLR: toll-like receptor
TNF-α: tumour necrosis factor-α
Treg: T regulatory cells
VAT: visceral adipose tissue
VCAM: vascular cell adhesion molecule
WAT: white adipose tissue

## References

[B1] Acedo S. C. Gambero S. Cunha F. G. Lorand-Metze I. Gambero A. (2013). Participation of leptin in the determination of the macrophage phenotype: an additional role in adipocyte and macrophage crosstalk. *In Vitro Cell. Dev. Biol. Anim.* 49 473–478. 10.1007/s11626-013-9629-x 23708919

[B2] Agrawal S. Gollapudi S. Su H. Gupta S. (2011). Leptin activates human B cells to secrete TNF-α, IL-6, and IL-10 via JAK2/STAT3 and p38MAPK/ERK1/2 signaling pathway. *J. Clin. Immunol.* 31 472–478. 10.1007/s10875-010-9507-1 21243519 PMC3132280

[B3] Ajeganova S. Andersson M. L. Hafström I. (2013). Association of obesity with worse disease severity in rheumatoid arthritis as well as with comorbidities: a long-term followup from disease onset. *Arthritis Care Res.* 65 78–87. 10.1002/acr.21710 22514159

[B4] Al-Hassi H. O. Bernardo D. Murugananthan A. U. Mann E. R. English N. R. Jones A. (2013). A mechanistic role for leptin in human dendritic cell migration: differences between ileum and colon in health and Crohn’s disease. *Mucosal Immunol.* 6 751–761. 10.1038/mi.2012.113 23168838 PMC3684777

[B5] Al-Suhaimi E. A. Shehzad A. (2013). Leptin, resistin and visfatin: the missing link between endocrine metabolic disorders and immunity. *Eur. J. Med. Res.* 18:12 10.1186/2047-783X-18-12 PMC3655867 23634778

[B6] Amarilyo G. Iikuni N. Liu A. Matarese G. La Cava A. (2014). Leptin enhances availability of apoptotic cell-derived self-antigen in systemic lupus erythematosus. *PLoS One* 9:e112826 10.1371/journal.pone.0112826 PMC4234630 25401752

[B7] Amarilyo G. Iikuni N. Shi F. D. Liu A. Matarese G. La Cava A. (2013). Leptin promotes lupus T-cell autoimmunity. *Clin. Immunol.* 149 530–533. 10.1016/j.clim.2013.09.002 24263282

[B8] Anders H. J. Rihl M. Heufelder A. Loch O. Schattenkirchner M. (1999). Leptin serum levels are not correlated with disease activity in patients with rheumatoid arthritis. *Metabolism* 48 745–748. 10.1016/S0026-0495(99)90174-9 10381149

[B9] Arrese M. Cabrera D. Kalergis A. M. Feldstein A. E. (2016). Innate immunity and inflammation in NAFLD/NASH. *Dig. Dis. Sci.* 61 1294–1303. 10.1007/s10620-016-4049-x 26841783 PMC4948286

[B10] Bähr I. Goritz V. Doberstein H. Gesine G. Hiller R. Rosenstock P. (2017). Diet-induced obesity is associated with an impaired NK cell function and an increased colon cancer incidence. *J. Nutr. Metab.* 2017:4297025 10.1155/2017/4297025 PMC5357539 28357137

[B11] Bao J. P. Chen W. P. Feng J. Hu P. F. Shi Z. L. Wu L. D. (2010). Leptin plays a catabolic role on articular cartilage. *Mol. Biol. Rep.* 37 3265–3272. 10.1007/s11033-009-9911-x 19876764

[B12] Batra A. Okur B. Glauben R. Erben U. Ihbe J. Stroh T. (2010). Leptin: a critical regulator of CD4^+^ T-cell polarization in vitro and in vivo. *Endocrinology* 151 56–62. 10.1210/en.2009-0565 19966187

[B13] Bennett B. D. Solar G. P. Yuan J. Q. Mathias J. Thomas G. R. Matthews W. (1996). A role for leptin and its cognate receptor in hematopoiesis. *Curr. Biol.* 6 1170–1180. 10.1016/S0960-9822(02)70684-2 8805376

[B14] Benomar Y. Naour N. Aubourg A. Bailleux V. Gertler A. Djiane J. (2006). Insulin and leptin induce Glut4 plasma membrane translocation and glucose uptake in a human neuronal cell line by a phosphatidylinositol 3-kinase-dependent mechanism. *Endocrinology* 147 2550–2556. 10.1210/en.2005-1464 16497805

[B15] Bhupathiraju S. Hu F. (2016). Epidemiology of obesity and diabetes and their cardiovascular complications. *Circ. Res.* 118 1723–1735. 10.1161/CIRCRESAHA.115.306825 27230638 PMC4887150

[B16] Bjorbaek C. Uotani S. Da Silva B. Flier J. S. Bjørbæk C. Uotani S. (1997). Divergent signaling capacities of the long and short isoforms of the leptin receptor. *J. Biol. Chem.* 272 32686–32695. 10.1074/jbc.272.51.32686 9405487

[B17] Boi S. K. Buchta C. M. Pearson N. A. Francis M. B. Meyerholz D. K. Grobe J. L. (2016). Obesity alters immune and metabolic profiles: new insight from obese-resistant mice on high-fat diet. *Obesity* 24 2140–2149. 10.1002/oby.21620 27515998 PMC5039085

[B18] Brotfain E. Hadad N. Shapira Y. Avinoah E. Zlotnik A. Raichel L. (2015). Neutrophil functions in morbidly obese subjects. *Clin. Exp. Immunol.* 181 156–163. 10.1111/cei.12631 25809538 PMC4469166

[B19] Bruno A. Conus S. Schmid I. Simon H.-U. (2005). Apoptotic pathways are inhibited by leptin receptor activation in neutrophils. *J. Immunol.* 174 8090–8096. 10.4049/jimmunol.174.12.8090 15944317

[B20] Busso N. So A. Chobaz-Peclat V. Morard C. Martinez-Soria E. Talabot-Ayer D. (2002). Leptin signaling deficiency impairs humoral and cellular immune responses and attenuates experimental arthritis. *J. Immunol.* 168 875–882. 10.4049/jimmunol.168.2.875 11777985

[B21] Byrne C. D. Targher G. (2015). NAFLD: a multisystem disease. *J. Hepatol.* 62 S47–S64. 10.1016/j.jhep.2014.12.012 25920090

[B22] Caldefie-Chezet F. Poulin A. Tridon A. Sion B. Vasson M. P. (2001). Leptin: a potential regulator of polymorphonuclear neutrophil bactericidal action? *J. Leukoc. Biol.* 69 414–418. 11261788

[B23] Caldefie-Chezet F. Poulin A. Vasson M.-P. (2003). Leptin regulates functional capacities of polymorphonuclear neutrophils. *Free Radic. Res.* 37 809–814. 10.1080/1071576031000097526 14567439

[B24] Cannon J. G. Sharma G. Sloan G. Dimitropoulou C. Baker R. R. Mazzoli A. (2014). Leptin regulates CD16 expression on human monocytes in a sex-specific manner. *Physiol. Rep.* 2:e12177 10.14814/phy2.12177 PMC4254102 25303952

[B25] Cao H. (2014). Adipocytokines in obesity and metabolic disease. *J. Endocrinol.* 220 T47–T59. 10.1530/JOE-13-0339 24403378 PMC3887367

[B26] Cao H. Lin J. Chen W. Xu G. Sun C. (2016). Baseline adiponectin and leptin levels in predicting an increased risk of disease activity in rheumatoid arthritis: a meta-analysis and systematic review. *Autoimmunity* 49 1–7. 10.1080/08916934.2016.1230847 27690205

[B27] Carlton E. D. Demas G. E. French S. S. (2012). Leptin, a neuroendocrine mediator of immune responses, inflammation, and sickness behaviors. *Horm. Behav.* 62 272–279. 10.1016/j.yhbeh.2012.04.010 22561456

[B28] Cassano S. Pucino V. La Rocca C. Procaccini C. De Rosa V. Marone G. (2014). Leptin modulates autophagy in human CD4^+^CD25^-^ conventional T cells. *Metabolism* 63 1272–1279. 10.1016/j.metabol.2014.06.010 25060689 PMC4180014

[B29] Chang S. F. Hsieh R. Z. Huang K. C. Chang C. A. Chiu F. Y. Kuo H. C. (2015). Upregulation of bone morphogenetic protein-2 synthesis and consequent collagen II expression in leptin-stimulated human chondrocytes. *PLoS One* 10:e0144252 10.1371/journal.pone.0144252 PMC4670096 26636769

[B30] Chatterjee S. Ganini D. Tokar E. J. Kumar A. Das S. Corbett J. (2013). Leptin is key to peroxynitrite-mediated oxidative stress and Kupffer cell activation in experimental non-alcoholic steatohepatitis. *J. Hepatol.* 58 778–784. 10.1016/j.jhep.2012.11.035 23207144 PMC3596459

[B31] Chimenti M. S. Triggianese P. Conigliaro P. Candi E. Melino G. Perricone R. (2015). The interplay between inflammation and metabolism in rheumatoid arthritis. *Cell Death Dis.* 6:e1887 10.1038/cddis.2015.246 PMC4650442 26379192

[B32] Chitturi S. Farrell G. Frost L. Kriketos A. Lin R. Fung C. (2002). Serum leptin in NASH correlates with hepatic steatosis but not fibrosis: a manifestation of lipotoxicity? *Hepatology* 36 403–409. 10.1053/jhep.2002.34738 12143049

[B33] Claycombe K. King L. E. Fraker P. J. (2008). A role for leptin in sustaining lymphopoiesis and myelopoiesis. *Proc. Natl. Acad. Sci. U.S.A.* 105 2017–2021. 10.1073/pnas.0712053105 18250302 PMC2538874

[B34] Cohen S. Danzaki K. MacIver N. J. (2017). Nutritional effects on T-cell immunometabolism. *Eur. J. Immunol.* 47 225–235. 10.1002/eji.201646423 28054344 PMC5342627

[B35] Conde J. Gomez R. Bianco G. Scotece M. Lear P. Dieguez C. (2011a). Expanding the adipokine network in cartilage: identification and regulation of novel factors in human and murine chondrocytes. *Ann. Rheum. Dis.* 70 551–559. 10.1136/ard.2010.132399 21216818

[B36] Conde J. Scotece M. Abella V. Gómez R. López V. Villar R. (2015). Identification of novel adipokines in the joint. Differential expression in healthy and osteoarthritis tissues. *PLoS One* 10:e0123601 10.1371/journal.pone.0123601 PMC4390373 25853553

[B37] Conde J. Scotece M. Gomez R. Lopez V. Gomez-Reino J. J. Gualillo O. (2011b). Adipokines and osteoarthritis: novel molecules involved in the pathogenesis and progression of disease. *Arthritis* 2011:203901 10.1155/2011/203901 PMC3200120 22046513

[B38] Conde J. Scotece M. López V. Abella V. Hermida M. Pino J. (2013). Differential expression of adipokines in infrapatellar fat pad (IPFP) and synovium of osteoarthritis patients and healthy individuals. *Ann. Rheum. Dis.* 73 631–633. 10.1136/annrheumdis-2013-204189 24099837

[B39] Conde J. Scotece M. López V. Gómez R. Lago F. Pino J. (2012). Adiponectin and leptin induce VCAM-1 expression in human and murine chondrocytes. *PLoS One* 7:e52533 10.1371/journal.pone.0052533 PMC3526577 23285079

[B40] Curat C. A. Miranville A. Sengenès C. Diehl M. Tonus C. Busse R. (2004). From blood monocytes to adipose tissue-resident macrophages: induction of diapedesis by human mature adipocytes. *Diabetes Metab. Res. Rev.* 53 1285–1292. 10.2337/diabetes.53.5.1285 15111498

[B41] Dayakar A. Chandrasekaran S. Veronica J. Maurya R. (2016). Leptin induces the phagocytosis and protective immune response in *Leishmania donovani* infected THP-1 cell line and human PBMCs. *Exp. Parasitol.* 160 54–59. 10.1016/j.exppara.2015.12.002 26688099

[B42] de Boer T. N. van Spil W. E. Huisman A. M. Polak A. A. Bijlsma J. W. Lafeber F. P. (2012). Serum adipokines in osteoarthritis; comparison with controls and relationship with local parameters of synovial inflammation and cartilage damage. *Osteoarthritis Cartilage* 20 846–853. 10.1016/j.joca.2012.05.002 22595228

[B43] De Rosa V. La Cava A. Matarese G. (2017). Metabolic pressure and the breach of immunological self-tolerance. *Nat. Immunol.* 18 1190–1196. 10.1038/ni.3851 29044230

[B44] De Rosa V. Procaccini C. Calì G. Pirozzi G. Fontana S. Zappacosta S. (2007). A key role of leptin in the control of regulatory T cell proliferation. *Immunity* 26 241–255. 10.1016/j.immuni.2007.01.011 17307705

[B45] DeFuria J. Belkina A. C. Jagannathan-Bogdan M. Snyder-Cappione J. Carr J. D. Nersesova Y. R. (2013). B cells promote inflammation in obesity and type 2 diabetes through regulation of T-cell function and an inflammatory cytokine profile. *Proc. Natl. Acad. Sci. U.S.A.* 110 5133–5138. 10.1073/pnas.1215840110 23479618 PMC3612635

[B46] Deiuliis J. A. (2016). MicroRNAs as regulators of metabolic disease: pathophysiologic significance and emerging role as biomarkers and therapeutics. *Int. J. Obes.* 40 88–101. 10.1038/ijo.2015.170 PMC4722234 26311337

[B47] Deng J. Liu Y. Yang M. Wang S. Zhang M. Wang X. (2012). Leptin exacerbates collagen-induced arthritis via enhancement of Th17 cell response. *Arthritis Rheum.* 64 3564–3573. 10.1002/art.34637 22833425

[B48] DePaoli A. M. (2014). Leptin in common obesity and associated disorders of metabolism. *J. Endocrinol.* 223 T71–T81. 10.1530/JOE-14-0258 24973357

[B49] Dib L. H. Ortega M. T. Melgarejo T. Chapes S. K. (2016). Establishment and characterization of DB-1: a leptin receptor-deficient murine macrophage cell line. *Cytotechnology* 68 921–933. 10.1007/s10616-015-9843-3 25599862 PMC4960141

[B50] Dixit V. D. Mielenz M. Taub D. D. Parvizi N. (2003). Leptin induces growth hormone secretion from peripheral blood mononuclear cells via a protein kinase C- and nitric oxide-dependent mechanism. *Endocrinology* 144 5595–5603. 10.1210/en.2003-0600 12970164

[B51] do Carmo L. S. Rogero M. M. Paredes-Gamero E. J. Nogueira-Pedro A. Xavier J. G. Cortez M. (2013). A high-fat diet increases interleukin-3 and granulocyte colony-stimulating factor production by bone marrow cells and triggers bone marrow hyperplasia and neutrophilia in Wistar rats. *Exp. Biol. Med.* 238 375–384. 10.1177/1535370213477976 23760003

[B52] Donath M. Y. (2014). Targeting inflammation in the treatment of type 2 diabetes: time to start. *Nat. Rev. Drug Discov.* 13 465–476. 10.1038/nrd4275 24854413

[B53] Donath M. Y. Shoelson S. E. (2011). Type 2 diabetes as an inflammatory disease. *Nat. Rev. Immunol.* 11 98–107. 10.1038/nri2925 21233852

[B54] Duarte G. V. Oliveira M. de F. Cardoso T. M. Follador I. Silva T. S. (2013). Association between obesity measured by different parameters and severity of psoriasis. *Int. J. Dermatol.* 52 177–181. 10.1111/j.1365-4632.2011.05270.x 22998685

[B55] Ducy P. Amling M. Takeda S. Priemel M. Schilling A. F. Beil F. T. (2000). Leptin inhibits bone formation through a hypothalamic relay: a central control of bone mass. *Cell* 100 197–207. 10.1016/S0092-8674(00)81558-5 10660043

[B56] Dumond H. Presle N. Terlain B. Mainard D. Loeuille D. Netter P. (2003). Evidence for a key role of leptin in osteoarthritis. *Arthritis Rheum.* 48 3118–3129. 10.1002/art.11303 14613274

[B57] Fan Q. Liu Z. Shen C. Li H. Ding J. Jin F. (2018). Microarray study of gene expression profile to identify new candidate genes involved in the molecular mechanism of leptin- induced knee joint osteoarthritis in rat. *Hereditas* 155:4 10.1186/s41065-017-0039-z PMC5496599 28690479

[B58] Farooqi I. S. Jebb S. A. Langmack G. Lawrence E. Cheetham C. H. Prentice A. M. (1999). Effects of recombinant leptin therapy in a child with congenital leptin deficiency. *N. Engl. J. Med.* 341 879–884. 10.1056/NEJM199909163411204 10486419

[B59] Farooqi I. S. Matarese G. Lord G. M. Keogh J. M. Lawrence E. Agwu C. (2002). Beneficial effects of leptin on obesity, T cell hyporesponsiveness, and neuroendocrine/metabolic dysfunction of human congenital leptin deficiency. *J. Clin. Invest.* 110 1093–1103. 10.1172/JCI15693 12393845 PMC150795

[B60] Fasshauer M. Blüher M. (2015). Adipokines in health and disease. *Trends Pharmacol. Sci.* 36 461–470. 10.1016/j.tips.2015.04.014 26022934

[B61] Favreau M. Menu E. Gaublomme D. Vanderkerken K. Faict S. Maes K. (2017). Leptin receptor antagonism of iNKT cell function: a novel strategy to combat multiple myeloma. *Leukemia* 31 2678–2685. 10.1038/leu.2017.146 28490813

[B62] Figenschau Y. Knutsen G. Shahazeydi S. Johansen O. Sveinbjörnsson B. (2001). Human articular chondrocytes express functional leptin receptors. *Biochem. Biophys. Res. Commun.* 287 190–197. 10.1006/bbrc.2001.5543 11549273

[B63] Findlay D. M. Atkins G. J. (2014). Osteoblast-chondrocyte interactions in osteoarthritis. *Curr. Osteoporos. Rep.* 12 127–134. 10.1007/s11914-014-0192-5 24458429 PMC3933767

[B64] Frasca D. Ferracci F. Diaz A. Romero M. Lechner S. Blomberg B. B. (2016). Obesity decreases B cell responses in young and elderly individuals. *Obesity* 24 615–625. 10.1002/oby.21383 26857091 PMC4769695

[B65] Fraser D. A. Thoen J. Reseland J. E. Førre O. Kjeldsen-Kragh J. (1999). Decreased CD4^+^ lymphocyte activation and increased interleukin-4 production in peripheral blood of rheumatoid arthritis patients after acute starvation. *Clin. Rheumatol.* 18 394–401. 10.1007/s100670050125 10524554

[B66] Frederich R. C. Hamann A. Anderson S. Löllmann B. Lowell B. B. Flier J. S. (1995). Leptin levels reflect body lipid content in mice: evidence for diet-induced resistance to leptin action. *Nat. Med.* 1 1311–1314. 10.1038/nm1295-1311 7489415

[B67] Frühbeck G. (2006). Intracellular signalling pathways activated by leptin. *Biochem. J.* 393 7–20. 10.1042/BJ20051578 16336196 PMC1383660

[B68] Fujita Y. Fujii T. Mimori T. Sato T. Nakamura T. Iwao H. (2014). Deficient leptin signaling ameliorates systemic lupus erythematosus lesions in MRL/Mp-Fas lpr mice. *J. Immunol.* 192 979–984. 10.4049/jimmunol.1301685 24391210 PMC3897175

[B69] Fujita Y. Yanagida H. Mimori T. Jin Z. X. Sakai T. Kawanami T. (2012). Prevention of fasting-mediated bone marrow atrophy by leptin administration. *Cell. Immunol.* 273 52–58. 10.1016/j.cellimm.2011.11.007 22196379

[B70] Gabay C. Dreyer M. G. Pellegrinelli N. Chicheportiche R. Meier C. A. (2001). Leptin directly induces the secretion of interleukin 1 receptor antagonist in human monocytes. *J. Clin. Endocrinol. Metab.* 86 783–791. 10.1210/jc.86.2.783 11158047

[B71] Gaber T. Strehl C. Buttgereit F. (2017). Metabolic regulation of inflammation. *Nat. Rev. Rheumatol.* 13 267–279. 10.1038/nrrheum.2017.37 28331208

[B72] Gatto M. Zen M. Ghirardello A. Bettio S. Bassi N. Iaccarino L. (2013). Emerging and critical issues in the pathogenesis of lupus. *Autoimmun. Rev.* 12 523–536. 10.1016/j.autrev.2012.09.003 23000207

[B73] Genovese M. C. Van den Bosch F. Roberson S. A. Bojin S. Biagini I. M. Ryan P. (2010). LY2439821, a humanized anti-interleukin-17 monoclonal antibody, in the treatment of patients with rheumatoid arthritis: a phase I randomized, double-blind, placebo-controlled, proof-of-concept study. *Arthritis Rheum.* 62 929–939. 10.1002/art.27334 20131262

[B74] Gerriets V. A. Danzaki K. Kishton R. J. Eisner W. Nichols A. G. Saucillo D. C. (2016). Leptin directly promotes T-cell glycolytic metabolism to drive effector T-cell differentiation in a mouse model of autoimmunity. *Eur. J. Immunol.* 46 1970–1983. 10.1002/eji.201545861 27222115 PMC5154618

[B75] Gerriets V. A. MacIver N. J. (2014). Role of T cells in malnutrition and obesity. *Front. Immunol.* 5:379 10.3389/fimmu.2014.00379 PMC4127479 25157251

[B76] Gomez R. Scotece M. Conde J. Gomez-Reino J. J. Lago F. Gualillo O. (2011). Adiponectin and leptin increase IL-8 production in human chondrocytes. *Ann. Rheum. Dis.* 70 2052–2054. 10.1136/ard.2010.145672 21622999

[B77] Griffin T. M. Huebner J. L. Kraus V. B. Guilak F. (2009). Extreme obesity due to impaired leptin signaling in mice does not cause knee osteoarthritis. *Arthritis Rheum.* 60 2935–2944. 10.1002/art.24854 19790050 PMC2829313

[B78] Grotta M. B. Squebola-Cola D. M. Toro A. A. Ribeiro M. A. Mazon S. B. Ribeiro J. D. (2013). Obesity increases eosinophil activity in asthmatic children and adolescents. *BMC Pulm. Med.* 13:39 10.1186/1471-2466-13-39 PMC3686646 23773659

[B79] Gruen M. L. Hao M. Piston D. W. Hasty A. H. (2007). Leptin requires canonical migratory signaling pathways for induction of monocyte and macrophage chemotaxis. *AJP Cell Physiol.* 293 C1481–C1488. 10.1152/ajpcell.00062.2007 17728393

[B80] Gupta S. Agrawal S. Gollapudi S. (2013). Increased activation and cytokine secretion in B cells stimulated with leptin in aged humans. *Immun. Ageing* 10:3 10.1186/1742-4933-10-3 PMC3557206 23343052

[B81] Gutierrez D. A. Hasty A. H. (2012). Haematopoietic leptin receptor deficiency does not affect macrophage accumulation in adipose tissue or systemic insulin sensitivity. *J. Endocrinol.* 212 343–351. 10.1530/JOE-11-0338 22194312 PMC3381898

[B82] Hizmetli S. Kisa M. Gokalp N. Bakici M. Z. (2007). Are plasma and synovial fluid leptin levels correlated with disease activity in rheumatoid arthritis? *Rheumatol. Int.* 27 335–338. 10.1007/s00296-006-0264-7 17102942

[B83] Howard J. K. Lord G. M. Matarese G. Vendetti S. Ghatei M. A. Ritter M. A. (1999). Leptin protects mice from starvation-induced lymphoid atrophy and increases thymic cellularity in ob/ob mice. *J. Clin. Invest.* 104 1051–1059. 10.1172/JCI6762 10525043 PMC408574

[B84] Huertas A. Phan C. Bordenave J. Tu L. Thuillet R. Le Hiress M. (2016). Regulatory T cell dysfunction in idiopathic, heritable and connective tissue-associated pulmonary arterial hypertension. *Chest* 149 1482–1493. 10.1016/j.chest.2016.01.004 26836928

[B85] Huh J. Y. Park Y. J. Ham M. Kim J. B. (2014). Crosstalk between adipocytes and immune cells in adipose tissue inflammation and metabolic dysregulation in obesity. *Mol. Cells* 37 365–371. 10.14348/molcells.2014.0074 24781408 PMC4044307

[B86] Hui W. Litherland G. J. Elias M. S. Kitson G. I. Cawston T. E. Rowan A. D. (2012). Leptin produced by joint white adipose tissue induces cartilage degradation via upregulation and activation of matrix metalloproteinases. *Ann. Rheum. Dis.* 71 455–462. 10.1136/annrheumdis-2011-200372 22072016

[B87] Ikejima K. Okumura K. Lang T. Honda H. Abe W. Yamashina S. (2005). The role of leptin in progression of non-alcoholic fatty liver disease. *Hepatol. Res.* 33 151–154. 10.1016/j.hepres.2005.09.024 16198623

[B88] Ikejima K. Takei Y. Honda H. Hirose M. Yoshikawa M. Zhang Y. J. (2002). Leptin receptor-mediated signaling regulates hepatic fibrogenesis and remodeling of extracellular matrix in the rat. *Gastroenterology* 122 1399–1410. 10.1053/gast.2002.32995 11984526

[B89] Inzaugarat M. E. De Matteo E. Baz P. Lucero D. Garcia C. C. Ballerga E. G. (2017). New evidence for the therapeutic potential of curcumin to treat nonalcoholic fatty liver disease in humans. *PLoS One* 12:e0172900 10.1371/journal.pone.0172900 PMC5336246 28257515

[B90] Jaedicke K. M. Roythorne A. Padget K. Todryk S. Preshaw P. M. Taylor J. J. (2013). Leptin up-regulates TLR2 in human monocytes. *J. Leukoc. Biol.* 93 561–571. 10.1189/jlb.1211606 23341537

[B91] Jahn J. Spielau M. Brandsch C. Stangl G. I. Delank K. S. Bähr I. (2015). Decreased NK cell functions in obesity can be reactivated by fat mass reduction. *Obesity* 23 2233–2241. 10.1002/oby.21229 26390898

[B92] Javor E. D. Ghany M. G. Cochran E. K. Oral E. A. DePaoli A. M. Premkumar A. (2005). Leptin reverses nonalcoholic steatohepatitis in patients with severe lipodystrophy. *Hepatology* 41 753–760. 10.1002/hep.20672 15791619

[B93] Jennbacken K. Ståhlman S. Grahnemo L. Wiklund O. Fogelstrand L. (2013). Glucose impairs B-1 cell function in diabetes. *Clin. Exp. Immunol.* 174 129–138. 10.1111/cei.12148 23731267 PMC3784220

[B94] Jones K. D. Thitiri J. Ngari M. Berkley J. A. (2014). Childhood malnutrition: toward an understanding of infection, inflammation, and antimicrobials. *Food Nutr. Bull.* 35(Suppl. 2), S64–S70. 10.1001/jama.2009.1266 25069296 PMC4257992

[B95] Kakuma T. Lee Y. Higa M. Wang Z. W. Pan W. Shimomura I. (2000). Leptin, troglitazone, and the expression of sterol regulatory element binding proteins in liver and pancreatic islets. *Proc. Natl. Acad. Sci. U.S.A.* 97 8536–8541. 10.1073/pnas.97.15.8536 10900012 PMC26983

[B96] Kalra S. P. (2009). Central leptin gene therapy ameliorates diabetes type 1 and 2 through two independent hypothalamic relays: a benefit beyond weight and appetite regulation. *Peptides* 30 1957–1963. 10.1016/j.peptides.2009.07.021 19647774 PMC2755606

[B97] Kamada Y. Takehara T. Hayashi N. (2008). Adipocytokines and liver disease. *J. Gastroenterol.* 43 811–822. 10.1007/s00535-008-2213-6 19012034

[B98] Kamp V. M. Langereis J. D. van Aalst C. W. van der Linden J. A. Ulfman L. H. Koenderman L. (2013). Physiological concentrations of leptin do not affect human neutrophils. *PLoS One* 8:e73170 10.1371/journal.pone.0073170 PMC3774682 24066032

[B99] Kato H. Ueki S. Kamada R. Kihara J. Yamauchi Y. Suzuki T. (2011). Leptin has a priming effect on eotaxin-induced human eosinophil chemotaxis. *Int. Arch. Allergy Immunol.* 155 335–344. 10.1159/000321195 21346363

[B100] Kavak K. S. Teter B. E. Hagemeier J. Zakalik K. Weinstock-Guttman B. Edwards K. (2015). Higher weight in adolescence and young adulthood is associated with an earlier age at multiple sclerosis onset. *Mult. Scler.* 21 858–865. 10.1177/1352458514555787 25392327

[B101] Kiguchi N. Maeda T. Kobayashi Y. Fukazawa Y. Kishioka S. (2009). Leptin enhances CC-chemokine ligand expression in cultured murine macrophage. *Biochem. Biophys. Res. Commun.* 384 311–315. 10.1016/j.bbrc.2009.04.121 19409880

[B102] Kim S. Y. Lim J. H. Choi S. W. Kim M. Kim S. T. Kim M. S. (2010). Preferential effects of leptin on CD4 T cells in central and peripheral immune system are critically linked to the expression of leptin receptor. *Biochem. Biophys. Res. Commun.* 394 562–568. 10.1016/j.bbrc.2010.03.019 20227394

[B103] Klöting N. Blüher M. (2014). Adipocyte dysfunction, inflammation and metabolic syndrome. *Rev. Endocr. Metab. Disord.* 15 277–287. 10.1007/s11154-014-9301-0 25344447

[B104] Koskinen A. Vuolteenaho K. Nieminen R. Moilanen T. Moilanen E. (2011). Leptin enhances MMP-1, MMP-3 and MMP-13 production in human osteoarthritic cartilage and correlates with MMP-1 and MMP-3 in synovial fluid from OA patients. *Clin. Exp. Rheumatol.* 29 57–64. 21345293

[B105] Koskinen-kolasa A. Vuolteenaho K. Korhonen R. Moilanen T. Moilanen E. (2016). Catabolic and proinflammatory effects of leptin in chondrocytes are regulated by suppressor of cytokine signaling-3. *Arthritis Res. Ther.* 215 1–13. 10.1186/s13075-016-1112-0 PMC5048607 27716333

[B106] Kulkarni R. N. Wang Z. L. Wang R. M. Hurley J. D. Smith D. M. Ghatei M. A. (1997). Leptin rapidly suppresses insulin release from insulinoma cells, rat and human islets and, in vivo, in mice. *J. Clin. Invest.* 100 2729–2736. 10.1172/JCI119818 9389736 PMC508476

[B107] Lam Q. L. Liu S. Cao X. Lu L. (2006). Involvement of leptin signaling in the survival and maturation of bone marrow-derived dendritic cells. *Eur. J. Immunol.* 36 3118–3130. 10.1002/eji.200636602 17125143

[B108] Lam Q. L. Wang S. Ko O. K. Kincade P. W. Lu L. (2010). Leptin signaling maintains B-cell homeostasis via induction of Bcl-2 and Cyclin D1. *Proc. Natl. Acad. Sci. U.S.A.* 107 13812–13817. 10.1073/pnas.1004185107 20643953 PMC2922219

[B109] Lamas A. Lopez E. Carrio R. Lopez D. M. (2016). Adipocyte and leptin accumulation in tumor-induced thymic involution. *Int. J. Mol. Med.* 37 133–138. 10.3892/ijmm.2015.2392 26530443

[B110] Lamas B. Goncalves-Mendes N. Nachat-Kappes R. Rossary A. Caldefie-Chezet F. Vasson M. P. (2013). Leptin modulates dose-dependently the metabolic and cytolytic activities of NK-92 cells. *J. Cell. Physiol.* 228 1202–1209. 10.1002/jcp.24273 23129404

[B111] Laue T. Wrann C. D. Hoffmann-Castendiek B. Pietsch D. Hübner L. Kielstein H. (2015). Altered NK cell function in obese healthy humans. *BMC Obes.* 2:1 10.1186/s40608-014-0033-1 PMC4511543 26217516

[B112] Lebovitz H. E. (2003). The relationship of obesity to the metabolic syndrome. *Int. J. Clin. Pract. Suppl.* 134 18–27. 12793594

[B113] Lee Y. Yu X. Gonzales F. Mangelsdorf D. J. Wang M.-Y. Richardson C. (2002). PPAR alpha is necessary for the lipopenic action of hyperleptinemia on white adipose and liver tissue. *Proc. Natl. Acad. Sci. U.S.A.* 99 11848–11853. 10.1073/pnas.182420899 12195019 PMC129357

[B114] Lee Y. H. Bae S. C. (2016). Circulating leptin level in rheumatoid arthritis and its correlation with disease activity: a meta-analysis. *Z. Rheumatol.* 75 1021–1027. 10.1007/s00393-016-0050-1 26820722

[B115] Lee Y. H. Song G. G. (2018). Association between circulating leptin levels and systemic lupus erythematosus: an updated meta-analysis. *Lupus* 27 428–435. 10.1177/0961203317725587 28795654

[B116] Li H. M. Zhang T. P. Leng R. X. Li X. P. Li X. M. Liu H. R. (2016). Emerging role of adipokines in systemic lupus erythematosus. *Immunol. Res.* 64 820–830. 10.1007/s12026-016-8808-8 27314594

[B117] Li K. Wei L. Huang Y. Wu Y. Su M. Pang X. (2016). Leptin promotes breast cancer cell migration and invasion via IL-18 expression and secretion. *Int. J. Oncol.* 48 2479–2487. 10.3892/ijo.2016.3483 27082857

[B118] Licinio J. Caglayan S. Ozata M. Yildiz B. O. de Miranda P. B. O’Kirwan F. (2004). Phenotypic effects of leptin replacement on morbid obesity, diabetes mellitus, hypogonadism, and behavior in leptin-deficient adults. *Proc. Natl. Acad. Sci. U.S.A.* 101 4531–4536. 10.1073/pnas.0308767101 15070752 PMC384781

[B119] Liu A. La Cava A. (2014). Epigenetic dysregulation in systemic lupus erythematosus. *Autoimmunity* 47 215–219. 10.3109/08916934.2013.844794 24128164

[B120] Liu Y. Yu Y. Matarese G. La Cava A. (2012). Cutting edge: fasting-induced hypoleptinemia expands functional regulatory T cells in systemic lupus erythematosus. *J. Immunol.* 188 2070–2073. 10.4049/jimmunol.1102835 22291185 PMC3288569

[B121] Lo C. Lam Q. L. Yang M. Ko K.-H. Sun L. Ma R. (2009). Leptin signaling protects NK cells from apoptosis during development in mouse bone marrow. *Cell. Mol. Immunol.* 6 353–360. 10.1038/cmi.2009.46 19887048 PMC4003218

[B122] Loeser R. F. Goldring S. R. Scanzello C. R. Goldring M. B. (2012). Osteoarthritis: a disease of the joint as an organ. *Arthritis Rheum.* 64 1697–1707. 10.1002/art.34453 22392533 PMC3366018

[B123] Lord G. M. Matarese G. Howard J. K. Baker R. J. Bloom S. R. Lechler R. I. (1998). Leptin modulates the T-cell immune response and reverses starvation-induced immunosuppression. *Nature* 394 897–901. 10.1038/29795 9732873

[B124] Lord G. M. Matarese G. Howard J. K. Bloom S. R. Lechler R. I. (2002). Leptin inhibits the anti-CD3-driven proliferation of peripheral blood T cells but enhances the production of proinflammatory cytokines. *J. Leukoc. Biol.* 72 330–338. 12149424

[B125] Lourenço E. V. Liu A. Matarese G. La Cava A. (2016). Leptin promotes systemic lupus erythematosus by increasing autoantibody production and inhibiting immune regulation. *Proc. Natl. Acad. Sci. U.S.A.* 113 10637–10642. 10.1073/pnas.1607101113 27588900 PMC5035847

[B126] Luan B. Goodarzi M. O. Phillips N. G. Guo X. Chen Y. D. Yao J. (2014). Leptin-mediated increases in catecholamine signaling reduce adipose tissue inflammation via activation of macrophage HDAC4. *Cell Metab.* 19 1058–1065. 10.1016/j.cmet.2014.03.024 24768298 PMC4207085

[B127] Ma L. Li D. Sookha M. R. Fang M. Guan Y. Sun X. (2015). Elevated serum leptin levels in patients with systemic lupus erythematosus. *Pharmazie* 70 720–723. 10.1691/ph.2015.5649 26790188

[B128] MacIver N. J. Michalek R. D. Rathmell J. C. (2013). Metabolic regulation of T lymphocytes. *Annu. Rev. Immunol.* 31 259–283. 10.1146/annurev-immunol-032712-095956 23298210 PMC3606674

[B129] Maingrette F. Renier G. (2003). Leptin increases lipoprotein lipase secretion by macrophages: involvement of oxidative stress and protein kinase C. *Diabetes Metab. Res. Rev.* 52 2121–2128. 10.2337/diabetes.52.8.2121 12882931

[B130] Mancuso P. (2004). Leptin augments alveolar macrophage leukotriene synthesis by increasing phospholipase activity and enhancing group IVC iPLA2 (cPLA2gamma) protein expression. *AJP Lung Cell. Mol. Physiol.* 287 L497–L502. 10.1152/ajplung.00010.2004 15145787

[B131] Mancuso P. Myers M. G. Goel D. Serezani C. H. O’Brien E. Goldberg J. (2012). Ablation of leptin receptor-mediated ERK activation impairs host defense against Gram-negative pneumonia. *J. Immunol.* 189 867–875. 10.4049/jimmunol.1200465 22685316 PMC3392451

[B132] Margiotta D. Navarini L. Vadacca M. Basta F. Vullo M. Pignataro F. (2016). Relationship between leptin and regulatory T cells in systemic lupus erythematosus?: preliminary results. *Eur. Rev. Med. Pharmacol. Sci.* 20 636–641. 26957264

[B133] Marques-Rocha J. L. Samblas M. Milagro F. I. Bressan J. Martínez J. A. Marti A. (2015). Noncoding RNAs, cytokines, and inflammation-related diseases. *FASEB J.* 29 3595–3611. 10.1096/fj.14-260323 26065857

[B134] Martínez-Carrillo B. E. Jarillo-Luna R. A. Campos-Rodríguez R. Valdés-Ramos R. Rivera-Aguilar V. (2015). Effect of diet and exercise on the peripheral immune system in young Balb/c mice. *Biomed Res. Int.* 2015:458470 10.1155/2015/458470 PMC4655039 26634209

[B135] Martín-Romero C. Santos-Alvarez J. Goberna R. Sánchez-Margalet V. (2000). Human leptin enhances activation and proliferation of human circulating monocytes. *Cell. Immunol.* 199 15–24. 10.1006/cimm.1999.1490 10675271

[B136] Matarese G. (2000). Leptin and the immune system: how nutritional status influences the immune response. *Eur. Cytokine Netw.* 11 7–13. 10.1519/JSC.0b013e3181bab493 10705294

[B137] Matarese G. Procaccini C. De Rosa V. Horvath T. L. La Cava A. (2010). Regulatory T cells in obesity: the leptin connection. *Trends Mol. Med.* 16 247–256. 10.1016/j.molmed.2010.04.002 20493774

[B138] Mathis D. Shoelson S. E. (2011). Immunometabolism: an emerging frontier. *Nat. Rev. Immunol.* 11 81–83. 10.1038/nri2922 21469396 PMC4784680

[B139] Mattioli B. Giordani L. Quaranta M. G. Viora M. (2009). Leptin exerts an anti-apoptotic effect on human dendritic cells via the PI3K-Akt signaling pathway. *FEBS Lett.* 583 1102–1106. 10.1016/j.febslet.2009.02.029 19250936

[B140] Mattioli B. Straface E. Matarrese P. Quaranta M. G. Giordani L. Malorni W. (2008). Leptin as an immunological adjuvant: enhanced migratory and CD8^+^ T cell stimulatory capacity of human dendritic cells exposed to leptin. *FASEB J.* 22 2012–2022. 10.1096/fj.07-098095 18218920

[B141] Maurya R. Bhattacharya P. Ismail N. Dagur P. K. Joshi A. B. Razdan K. (2016). Differential role of leptin as an immunomodulator in controlling visceral Leishmaniasis in normal and leptin-deficient mice. *Am. J. Trop. Med. Hyg.* 95 109–119. 10.4269/ajtmh.15-0804 27114296 PMC4944674

[B142] McInnes I. (2011). The pathogenesis of rheumatoid arthritis. *N. Engl. J. Med.* 365 2205–2219. 10.7748/phc2011.11.21.9.29.c8797 22150039

[B143] Meier C. A. Chicheportiche R. Dreyer M. Dayer J. M. (2003). IP-10, but not RANTES, is upregulated by leptin in monocytic cells. *Cytokine* 21 43–47. 10.1016/S1043-4666(02)00491-X 12668159

[B144] Mittendorfer B. Horowitz J. F. DePaoli A. M. McCamish M. A. Patterson B. W. Klein S. (2011). Recombinant human leptin treatment does not improve insulin action in obese subjects with type 2 diabetes. *Diabetes Metab. Res. Rev.* 60 1474–1477. 10.2337/db10-1302 PMC3292320 21411512

[B145] Montecucco F. Bianchi G. Gnerre P. Bertolotto M. Dallegri F. Ottonello L. (2006). Induction of neutrophil chemotaxis by leptin: crucial role for p38 and Src kinases. *Ann. N. Y. Acad. Sci.* 1069 463–471. 10.1196/annals.1351.045 16855174

[B146] Moon H.-S. Matarese G. Brennan A. M. Chamberland J. P. Liu X. Fiorenza C. G. (2011). Efficacy of metreleptin in obese patients with type 2 diabetes: cellular and molecular pathways underlying leptin tolerance. *Diabetes Metab. Res. Rev.* 60 1647–1656. 10.2337/db10-1791 PMC3114380 21617185

[B147] Moraes-Vieira P. M. Larocca R. A. Bassi E. J. Peron J. P. Andrade-Oliveira V. Wasinski F. (2014). Leptin deficiency impairs maturation of dendritic cells and enhances induction of regulatory T and Th17 cells. *Eur. J. Immunol.* 44 794–806. 10.1002/eji.201343592 24271843 PMC4973395

[B148] Morteza A. Nakhjavani M. Asgarani F. Ghaneei A. Esteghamati A. Mirmiranpour H. (2013). The lost correlation between leptin and CRP in type 2 diabetes. *Eur. Cytokine Netw.* 24 53–59. 10.1684/ecn.2013.0329 23674518

[B149] Morton G. J. Gelling R. W. Niswender K. D. Morrison C. D. Rhodes C. J. Schwartz M. W. (2005). Leptin regulates insulin sensitivity via phosphatidylinositol-3-OH kinase signaling in mediobasal hypothalamic neurons. *Cell Metab.* 2 411–420. 10.1016/j.cmet.2005.10.009 16330326

[B150] Münzberg H. Morrison C. D. (2015). Structure, production and signaling of leptin. *Metabolism* 64 13–23. 10.1016/j.metabol.2014.09.010 25305050 PMC4267896

[B151] Mutabaruka M.-S. Aoulad Aissa M. Delalandre A. Lavigne M. Lajeunesse D. (2010). Local leptin production in osteoarthritis subchondral osteoblasts may be responsible for their abnormal phenotypic expression. *Arthritis Res. Ther.* 12:R20 10.1186/ar2925 PMC2875652 20141628

[B152] Najib S. Sánchez-Margalet V. (2002). Human leptin promotes survival of human circulating blood monocytes prone to apoptosis by activation of p42/44 MAPK pathway. *Cell. Immunol.* 220 143–149. 10.1016/S0008-8749(03)00027-3 12657249

[B153] Nakamura Y. Sanematsu K. Ohta R. Shirosaki S. Koyano K. Nonaka K. (2008). Diurnal variation of human sweet taste recognition thresholds is correlated with plasma leptin levels. *Diabetes Metab. Res. Rev.* 57 2661–2665. 10.2337/db07-1103 PMC2551675 18633111

[B154] Namkoong C. Kim M. S. Jang P. G. Han S. M. Park H. S. Koh E. H. (2005). Enhanced hypothalamic AMP-activated protein kinase activity contributes to hyperphagia in diabetic rats. *Diabetes Metab. Res. Rev.* 54 63–68. 10.2337/diabetes.54.1.63 15616011

[B155] Nave H. Mueller G. Siegmund B. Jacobs R. Stroh T. Schueler U. (2008). Resistance of Janus kinase-2 dependent leptin signaling in natural killer (NK) Cells: a novel mechanism of NK cell dysfunction in diet-induced obesity. *Endocrinology* 149 3370–3378. 10.1210/en.2007-1516 18356278

[B156] Naylor C. Burgess S. Madan R. Buonomo E. Razzaq K. Ralston K. (2014). Leptin receptor mutation results in defective neutrophil recruitment to the colon during *Entamoeba histolytica* infection. *mBio* 5:e02046–14 10.1128/mBio.02046-14 PMC4271549 25516614

[B157] Nikolajczyk B. S. (2010). B cells as under-appreciated mediators of non-auto-immune inflammatory disease. *Cytokine* 50 234–242. 10.1016/j.cyto.2010.02.022 20382544 PMC2917985

[B158] Nugent M. (2016). MicroRNAs: exploring new horizons in osteoarthritis. *Osteoarthritis Cartilage* 24 573–580. 10.1016/j.joca.2015.10.018 26576510

[B159] Olama S. M. Senna M. K. Elarman M. (2012). Synovial/Serum leptin ratio in rheumatoid arthritis: the association with activity and erosion. *Rheumatol. Int.* 32 683–690. 10.1007/s00296-010-1698-5 21140264

[B160] Oner S. Volkan O. Oner C. Mengi A. Direskeneli H. Da T. (2015). Serum leptin levels do not correlate with disease activity in rheumatoid arthritis. *Acta Reumatol. Port.* 40 50–54. 25342093

[B161] Oral E. A. Simha V. Ruiz E. Andewelt A. Premkumar A. Snell P. (2002). Leptin-replacement therapy for lipodystrophy. *N. Engl. J. Med.* 346 570–578. 10.1056/NEJMoa012437 11856796

[B162] Orlova E. G. Shirshev S. V. (2014). Role of leptin and ghrelin in induction of differentiation of IL-17-producing and T-regulatory cells. *Bull. Exp. Biol. Med.* 156 819–822. 10.1007/s10517-014-2459-3 24824706

[B163] O’Rourke L. Yeaman S. J. Shepherd P. R. (2001). Insulin and leptin acutely regulate cholesterol ester metabolism in macrophages by novel signaling pathways. *Diabetes Metab. Res. Rev.* 50 955–961. 10.2337/diabetes.50.5.955 11334438

[B164] Otero M. Gomez Reino J. J. Gualillo O. (2003). Synergistic induction of nitric oxide synthase type II: in vitro effect of leptin and interferon-gamma in human chondrocytes and ATDC5 chondrogenic cells. *Arthritis Rheum.* 48 404–409. 10.1002/art.10811 12571850

[B165] Otero M. Lago R. Gomez R. Lago F. Dieguez C. Gómez-Reino J. J. (2006). Changes in plasma levels of fat-derived hormones adiponectin, leptin, resistin and visfatin in patients with rheumatoid arthritis. *Ann. Rheum. Dis.* 65 1198–1201. 10.1136/ard.2005.046540 16414972 PMC1798289

[B166] Otero M. Lago R. Gómez R. Lago F. Gomez-Reino J. J. Gualillo O. (2007). Phosphatidylinositol 3-kinase, MEK-1 and p38 mediate leptin/interferon-gamma synergistic NOS type II induction in chondrocytes. *Life Sci.* 81 1452–1460. 10.1016/j.lfs.2007.09.007 17935739

[B167] Otero M. Lago R. Lago F. Reino J. J. Gualillo O. (2005). Signalling pathway involved in nitric oxide synthase type II activation in chondrocytes: synergistic effect of leptin with interleukin-1. *Arthritis Res. Ther.* 7 R581–R591. 10.1186/ar1708 15899045 PMC1174950

[B168] Otvos L. Shao W.-H. Vanniasinghe A. S. Amon M. A. Csilla Holub M. Kovalszky I. (2011). Toward understanding the role of leptin and leptin receptor antagonism in preclinical models of rheumatoid arthritis. *Peptides* 32 1567–1574. 10.1016/j.peptides.2011.06.015 21723351

[B169] Ozata M. Uçkaya G. Beyhan Z. Ozdemir I. C. (1999). Plasma leptin levels in male patients with idiopathic central diabetes insipidus. *J. Endocrinol. Invest.* 22 451–454. 10.1007/BF03343589 10435855

[B170] Pearson M. J. Herndler-Brandstetter D. Tariq M. A. Nicholson T. A. Philp A. M. Smith H. L. (2017). IL-6 secretion in osteoarthritis patients is mediated by chondrocyte-synovial fibroblast cross-talk and is enhanced by obesity. *Sci. Rep.* 7:3451 10.1038/s41598-017-03759-w PMC5471184 28615667

[B171] Pelleymounter M. A. Cullen M. J. Baker M. B. Hecht R. Winters D. Boone T. (1995). Effects of the obese gene product on body weight regulation in ob/ob mice. *Science* 269 540–543. 10.1126/science.7624776 7624776

[B172] Pérez-Pérez A. Vilariño-García T. Fernández-Riejos P. Martín-González J. Segura-Egea J. J. Sánchez-Margalet V. (2017). Role of leptin as a link between metabolism and the immune system. *Cytokine Growth Factor Rev.* 35 71–84. 10.1016/j.cytogfr.2017.03.001 28285098

[B173] Petersen K. F. Oral E. A. Dufour S. Befroy D. Ariyan C. Yu C. (2002). Leptin reverses insulin resistance and hepatic steatosis in patients with severe lipodystrophy. *J. Clin. Invest.* 109 1345–1350. 10.1172/JCI200215001 12021250 PMC150981

[B174] Polyzos S. A. Kountouras J. Mantzoros C. S. (2015). Leptin in nonalcoholic fatty liver disease: a narrative review. *Metabolism* 64 60–78. 10.1016/j.metabol.2014.10.012 25456097

[B175] Popa C. Netea M. G. Radstake T. R. D. van Riel P. L. Barrera P. van der Meer J. W. (2005). Markers of inflammation are negatively correlated with serum leptin in rheumatoid arthritis. *Ann. Rheum. Dis.* 64 1195–1198. 10.1136/ard.2004.032243 15731289 PMC1755600

[B176] Procaccini C. De Rosa V. Galgani M. Carbone F. Cassano S. Greco D. (2012). Leptin-induced mTOR activation defines a specific molecular and transcriptional signature controlling CD4+ effector T cell responses. *J. Immunol.* 189 2941–2953. 10.4049/jimmunol.1200935 22904304

[B177] Procaccini C. La Rocca C. Carbone F. De Rosa V. Galgani M. Matarese G. (2017). Leptin as immune mediator: interaction between neuroendocrine and immune system. *Dev. Comp. Immunol.* 66 120–129. 10.1016/j.dci.2016.06.006 27288847

[B178] Rahmati M. Mobasheri A. Mozafari M. (2016). Inflammatory mediators in osteoarthritis: a critical review of the state-of-the-art, current prospects, and future challenges. *Bone* 85 81–90. 10.1016/j.bone.2016.01.019 26812612

[B179] Ramirez O. Garza K. M. (2014). Leptin deficiency in vivo enhances the ability of splenic dendritic cells to activate T cells. *Int. Immunol.* 26 627–636. 10.1093/intimm/dxu067 24966213 PMC4201843

[B180] Reis B. S. Lee K. Fanok M. H. Mascaraque C. Amoury M. Cohn L. B. (2015). Leptin receptor signaling in T cells is required for Th17 differentiation. *J. Immunol.* 194 5253–5260. 10.4049/jimmunol.1402996 25917102 PMC4433844

[B181] Rodríguez L. Graniel J. Ortiz R. (2007). Effect of leptin on activation and cytokine synthesis in peripheral blood lymphocytes of malnourished infected children. *Clin. Exp. Immunol.* 148 478–485. 10.1111/j.1365-2249.2007.03361.x 17355247 PMC1941936

[B182] Rosenbaum M. Leibel R. L. (2014). Role of leptin in energy homeostasis in humans. *J. Endocrinol.* 223 T83–T96. 10.1530/JOE-14-0358 25063755 PMC4454393

[B183] Sanchez-Margalet V. Martin-Romero C. (2001). Human leptin signaling in human peripheral blood mononuclear cells: activation of the JAK-STAT pathway. *Cell. Immunol.* 211 30–36. 10.1006/cimm.2001.1815 11585385

[B184] Sánchez-Pozo C. Rodriguez-Baño J. Domínguez-Castellano A. Muniain M. A. Goberna R. Sánchez-Margalet V. (2003). Leptin stimulates the oxidative burst in control monocytes but attenuates the oxidative burst in monocytes from HIV-infected patients. *Clin. Exp. Immunol.* 134 464–469. 10.1111/j.1365-2249.2003.02321.x 14632752 PMC1808878

[B185] Santos-Alvarez J. Goberna R. Sánchez-Margalet V. (1999). Human leptin stimulates proliferation and activation of human circulating monocytes. *Cell. Immunol.* 194 6–11. 10.1006/cimm.1999.1490 10357875

[B186] Saucillo D. C. Gerriets V. A. Sheng J. Rathmell J. C. Maciver N. J. Stedman S. W. (2014). Leptin metabolically licenses T cells for activation to link nutrition and immunity. *J. Immunol.* 192 136–144. 10.4049/jimmunol.1301158 24273001 PMC3872216

[B187] Saxena N. K. Ikeda K. Rockey D. C. Friedman S. L. (2002). Leptin in hepatic fibrosis: evidence for increased collagen production in stellate cells and lean littermates of ob/ob mice. *Hepatology* 35 762–771. 10.1053/jhep.2002.32029 11915021 PMC2935193

[B188] Schultze S. M. Hemmings B. A. Niessen M. Tschopp O. (2012). PI3K/AKT, MAPK and AMPK signalling: protein kinases in glucose homeostasis. *Expert Rev. Mol. Med.* 14:e1 10.1017/S1462399411002109 22233681

[B189] Scotece M. Conde J. López V. Lago F. Pino J. Gómez-Reino J. J. (2014). Adiponectin and leptin: new targets in inflammation. *Basic Clin. Pharmacol. Toxicol.* 114 97–102. 10.1111/bcpt.12109 23834523

[B190] Scotece M. Pérez T. Conde J. Abella V. López V. Pino J. (2017). Adipokines induce pro-inflammatory factors in activated Cd4+ T cells from osteoarthritis patient. *J. Orthop. Res.* 35 1299–1303. 10.1002/jor.23377 27472907

[B191] Seol D. McCabe D. J. Choe H. Zheng H. Yu Y. Jang K. (2012). Chondrogenic progenitor cells respond to cartilage injury. *Arthritis Rheum.* 64 3626–3637. 10.1002/art.34613 22777600 PMC4950521

[B192] Silha J. V. Krsek M. Skrha J. V. Sucharda P. Nyomba B. L. Murphy L. J. (2003). Plasma resistin, adiponectin and leptin levels in lean and obese subjects: correlations with insulin resistence. *Eur. J. Endocrinol.* 149 331–335. 10.1530/eje.0.1490331 14514348

[B193] Smolen J. S. Aletaha D. McInnes I. B. (2016). Rheumatoid arthritis. *Lancet* 388 2023–2038. 10.1016/S0140-6736(16)30173-8 27156434

[B194] Sun Z. Dragon S. Becker A. Gounni A. S. (2013). Leptin inhibits neutrophil apoptosis in children via ERK/NF-κB-dependent pathways. *PLoS One* 8:e55249 10.1371/journal.pone.0055249 PMC3561393 23383125

[B195] Suzukawa M. Nagase H. Ogahara I. Han K. Tashimo H. Shibui A. (2011). Leptin enhances survival and induces migration, degranulation, and cytokine synthesis of human basophils. *J. Immunol.* 186 5254–5260. 10.4049/jimmunol.1004054 21421855

[B196] Tacke F. Yoneyama H. (2013). From NAFLD to NASH to fibrosis to HCC: role of dendritic cell populations in the liver. *Hepatology* 58 494–496. 10.1002/hep.26405 23519833

[B197] Tajiri K. Shimizu Y. (2012). Role of NKT cells in the pathogenesis of NAFLD. *Int. J. Hepatol.* 2012:850836 10.1155/2012/850836 PMC3335183 22577564

[B198] Tanaka M. Suganami T. Kim-Saijo M. Toda C. Tsuiji M. Ochi K. (2011). Role of central leptin signaling in the starvation-induced alteration of B-cell development. *J. Neurosci.* 31 8373–8380. 10.1523/JNEUROSCI.6562-10.2011 21653842 PMC6623333

[B199] Targońska-Stępniak B. Majdan M. Dryglewska M. (2008). Leptin serum levels in rheumatoid arthritis patients: relation to disease duration and activity. *Rheumatol. Int.* 28 585–591. 10.1007/s00296-007-0480-9 17968549

[B200] Taylor A. K. Cao W. Vora K. P. De La Cruz J. Shieh W. J. Zaki S. R. (2013). Protein energy malnutrition decreases immunity and increases susceptibility to influenza infection in mice. *J. Infect. Dis.* 207 501–510. 10.1093/infdis/jis527 22949306 PMC11341849

[B201] Tchang B. G. Shukla A. P. Aronne L. J. (2015). Metreleptin and generalized lipodystrophy and evolving therapeutic perspectives. *Expert Opin. Biol. Ther.* 15 1061–1075. 10.1517/14712598.2015.1052789 26063386

[B202] Tian G. Liang J.-N. Wang Z.-Y. Zhou D. (2014). Emerging role of leptin in rheumatoid arthritis. *Clin. Exp. Immunol.* 177 557–570. 10.1111/cei.12372 24802245 PMC4137840

[B203] Tian Z. Chen Y. Gao B. (2013). Natural killer cells in liver disease. *Hepatology* 57 1654–1662. 10.1002/hep.26115 23111952 PMC3573257

[B204] Tian Z. Sun R. Wei H. Gao B. (2002). Impaired natural killer (NK) cell activity in leptin receptor deficient mice: leptin as a critical regulator in NK cell development and activation. *Biochem. Biophys. Res. Commun.* 298 297–302. 10.1016/S0006-291X(02)02462-2 12413939

[B205] Tilg H. Moschen A. R. (2006). Adipocytokines: mediators linking adipose tissue, inflammation and immunity. *Nat. Rev. Immunol.* 6 772–783. 10.1038/nri1937 16998510

[B206] Tiniakos D. G. Vos M. B. Brunt E. M. (2010). Nonalcoholic fatty liver disease: pathology and pathogenesis. *Annu. Rev. Pathol. Mech. Dis.* 5 145–171. 10.1146/annurev-pathol-121808-102132 20078219

[B207] Toussirot É. Michel F. Binda D. Dumoulin G. (2015). The role of leptin in the pathophysiology of rheumatoid arthritis. *Life Sci.* 140 29–36. 10.1016/j.lfs.2015.05.001 26025594

[B208] Tsiotra P. C. Boutati E. Dimitriadis G. Raptis S. A. (2013). High insulin and leptin increase resistin and inflammatory cytokine production from human mononuclear cells. *Biomed Res. Int.* 2013:487081 10.1155/2013/487081 PMC3591160 23484124

[B209] Tsiotra P. C. Pappa V. Raptis S. A. Tsigos C. (2000). Expression of the long and short leptin receptor isoforms in peripheral blood mononuclear cells: implications for leptin’s actions. *Metabolism* 49 1537–1541. 10.1053/meta.2000.18519 11145113

[B210] Tsochatzis E. A. Papatheodoridis G. V. Archimandritis A. J. (2009). Adipokines in nonalcoholic steatohepatitis: from pathogenesis to implications in diagnosis and therapy. *Mediators Inflamm.* 2009:831670 10.1155/2009/831670 PMC2694309 19753129

[B211] Ubags N. D. Vernooy J. H. Burg E. Hayes C. Bement J. Dilli E. (2014). The role of leptin in the development of pulmonary neutrophilia in infection and Acute Lung Injury. *Crit. Care Med.* 42 143–151. 10.1097/CCM.0000000000000048 PMC3947045 24231757

[B212] Vestweber D. (2015). How leukocytes cross the vascular endothelium. *Nat. Rev. Immunol.* 15 692–704. 10.1038/nri3908 26471775

[B213] Vieira-Potter V. J. (2014). Inflammation and macrophage modulation in adipose tissues. *Cell. Microbiol.* 16 1484–1492. 10.1111/cmi.12336 25073615

[B214] Voloshyna I. Mounessa J. Carsons S. E. Reiss A. B. (2016). Effect of inhibition of interleukin-12/23 by ustekinumab on the expression of leptin and leptin receptor in human THP-1 macrophages. *Clin. Exp. Dermatol.* 41 308–311. 10.1111/ced.12699 26095599 PMC4685020

[B215] Vuolteenaho K. Koskinen A. Kukkonen M. Nieminen R. Päivärinta U. Moilanen T. (2009). Leptin enhances synthesis of proinflammatory mediators in human osteoarthritic cartilage–mediator role of NO in leptin-induced PGE2, IL-6, and IL-8 production. *Mediators Inflamm.* 2009:345838 10.1155/2009/345838 PMC2726438 19688109

[B216] Wagner N. M. Brandhorst G. Czepluch F. Lankeit M. Eberle C. Herzberg S. (2013). Circulating regulatory T cells are reduced in obesity and may identify subjects at increased metabolic and cardiovascular risk. *Obesity* 21 461–468. 10.1002/oby.20087 23592653

[B217] Wang J. Leclercq I. Brymora J. M. Xu N. Ramezani-Moghadam M. London R. M. (2009). Kupffer cells mediate leptin-induced liver fibrosis. *Gastroenterology* 137 713–723. 10.1053/j.gastro.2009.04.011 19375424 PMC2757122

[B218] Wang S. Baidoo S. E. Liu Y. Zhu C. Tian J. Ma J. (2013). T cell-derived leptin contributes to increased frequency of T helper type 17 cells in female patients with Hashimoto’s thyroiditis. *Clin. Exp. Immunol.* 171 63–68. 10.1111/j.1365-2249.2012.04670.x 23199324 PMC3530096

[B219] Wang X. Qiao Y. Yang L. Song S. Han Y. Tian Y. (2017). Leptin levels in patients with systemic lupus erythematosus inversely correlate with regulatory T cell frequency. *Lupus* 26 1401–1406. 10.1177/0961203317703497 28409523

[B220] Winer D. A. Winer S. Shen L. Wadia P. P. Yantha J. Paltser G. (2011). B cells promote insulin resistance through modulation of T cells and production of pathogenic IgG antibodies. *Nat. Med.* 17 610–617. 10.1038/nm.2353 21499269 PMC3270885

[B221] Wolsk E. Mygind H. Grondahl T. S. Pedersen B. K. van Hall G. (2011). The role of leptin in human lipid and glucose metabolism: the effects of acute recombinant human leptin infusion in young healthy males. *Am. J. Clin. Nutr.* 94 1533–1544. 10.3945/ajcn.111.012260 22071709

[B222] Wong C. K. Cheung P. F. Lam C. W. (2007). Leptin-mediated cytokine release and migration of eosinophils: implications for immunopathophysiology of allergic inflammation. *Eur. J. Immunol.* 37 2337–2348. 10.1002/eji.200636866 17634954

[B223] Wrann C. D. Laue T. Hübner L. Kuhlmann S. Jacobs R. Goudeva L. (2012). Short-term and long-term leptin exposure differentially affect human natural killer cell immune functions. *Am. J. Physiol. Endocrinol. Metab.* 302 E108–E116. 10.1152/ajpendo.00057.2011 21952038

[B224] Xu R. Huang H. Zhang Z. Wang F.-S. (2014). The role of neutrophils in the development of liver diseases. *Cell. Mol. Immunol.* 11 224–231. 10.1038/cmi.2014.2 24633014 PMC4085492

[B225] Xu W. D. Zhang M. Zhang Y. J. Liu S. S. Pan H. F. Ye D. Q. (2014). Association between leptin and systemic lupus erythematosus. *Rheumatol. Int.* 34 559–563. 10.1007/s00296-013-2774-4 23666119

[B226] Yadav A. Jyoti P. Jain S. K. Bhattacharjee J. (2011). Correlation of adiponectin and leptin with insulin resistance: a pilot study in healthy north Indian population. *Indian J. Clin. Biochem.* 26 193–196. 10.1007/s12291-011-0119-1 22468049 PMC3107404

[B227] Yoshino T. Kusunoki N. Tanaka N. Kaneko K. Kusunoki Y. Endo H. (2011). Elevated serum levels of resistin, leptin, and adiponectin are associated with C-reactive protein and also other clinical conditions in rheumatoid arthritis. *Intern. Med.* 50 269–275. 10.2169/internalmedicine.50.4306 21325757

[B228] Yu Y. Liu Y. Shi F.-D. Zou H. Matarese G. La Cava A. (2013). Cutting edge: leptin-induced RORγt expression in CD4+ T cells promotes Th17 responses in systemic lupus erythematosus. *J. Immunol.* 190 3054–3058. 10.4049/jimmunol.1203275 23447682 PMC3608794

[B229] Zarkesh-Esfahani H. Pockley A. G. Wu Z. Hellewell P. G. Weetman A. P. Ross R. J. (2004). Leptin indirectly activates human neutrophils via induction of TNF-α. *J. Immunol.* 172 1809–1814. 10.4049/jimmunol.172.3.1809 14734764

[B230] Zhang Y. Liu J. Yao J. Ji G. Qian L. Wang J. (2014). Obesity: pathophysiology and intervention. *Nutrients* 6 5153–5183. 10.3390/nu6115153 25412152 PMC4245585

[B231] Zhang Y. Proenca R. Maffei M. Barone M. Leopold L. Friedman J. M. (1994). Positional cloning of the mouse obese gene and its human homologue. *Nature* 372 425–432. 10.1038/372425a0 7984236

[B232] Zhao F.-Q. Keating A. (2007). Functional properties and genomics of glucose transporters. *Curr. Genomics* 8 113–128. 10.2174/138920207780368187 18660845 PMC2435356

[B233] Zhao X. Dong Y. Zhang J. Li D. Hu G. Yao J. (2016). Leptin changes differentiation fate and induces senescence in chondrogenic progenitor cells. *Cell Death Dis.* 7:e2188 10.1038/cddis.2016.68 PMC4855655 27077804

[B234] Zhao Y. Sun R. You L. Gao C. Tian Z. (2003). Expression of leptin receptors and response to leptin stimulation of human natural killer cell lines. *Biochem. Biophys. Res. Commun.* 300 247–252. 10.1016/S0006-291X(02)02838-3 12504075

[B235] Zheng C. Yang Q. Cao J. Xie N. Liu K. Shou P. (2016). Local proliferation initiates macrophage accumulation in adipose tissue during obesity. *Cell Death Dis.* 7:e2167 10.1038/cddis.2016.54 PMC4823955 27031964

[B236] Zhou B. Li H. Shi J. (2017). miR-27 inhibits the NF-κB signaling pathway by targeting leptin in osteoarthritic chondrocytes. *Int. J. Mol. Med.* 40 523–530. 10.3892/ijmm.2017.3021 28627582

[B237] Zhou Y. Rui L. (2014). Leptin signaling and leptin resistance. *Front. Med.* 7 207–222. 10.1007/s11684-013-0263-5 PMC4069066 23580174

[B238] Zhou Y. Yu X. Chen H. Sjöberg S. Roux J. Zhang L. (2015). Leptin deficiency shifts mast cells toward anti-inflammatory actions and protects mice from obesity and diabetes by polarizing M2 macrophages. *Cell Metab.* 22 1045–1058. 10.1016/j.cmet.2015.09.013 26481668 PMC4670585

